# Biomedical applications, perspectives and tag design concepts in the cell – silent Raman window†

**DOI:** 10.1039/d3cb00217a

**Published:** 2024-02-12

**Authors:** Martha Z. Vardaki, Vasilis G. Gregoriou, Christos L. Chochos

**Affiliations:** a Institute of Chemical Biology, National Hellenic Research Foundation, 48 Vassileos Constantinou Avenue Athens 11635 Greece chochos@eie.gr; b Advent Technologies SA, Stadiou Street, Platani Rio Patras 26504 Greece

## Abstract

Spectroscopic studies increasingly employ Raman tags exhibiting a signal in the cell – silent region of the Raman spectrum (1800–2800 cm^−1^), where bands arising from biological molecules are inherently absent. Raman tags bearing functional groups which contain a triple bond, such as alkyne and nitrile or a carbon–deuterium bond, have a distinct vibrational frequency in this region. Due to the lack of spectral background and cell-associated bands in the specific area, the implementation of those tags can help overcome the inherently poor signal-to-noise ratio and presence of overlapping Raman bands in measurements of biological samples. The cell – silent Raman tags allow for bioorthogonal imaging of biomolecules with improved chemical contrast and they have found application in analyte detection and monitoring, biomarker profiling and live cell imaging. This review focuses on the potential of the cell – silent Raman region, reporting on the tags employed for biomedical applications using variants of Raman spectroscopy.

## Abstract 2

This comprehensive review article discusses the potential of the cell – silent Raman region, reporting on the tags employed for biomedical applications using variants of Raman spectroscopy.

## Graphical abstract

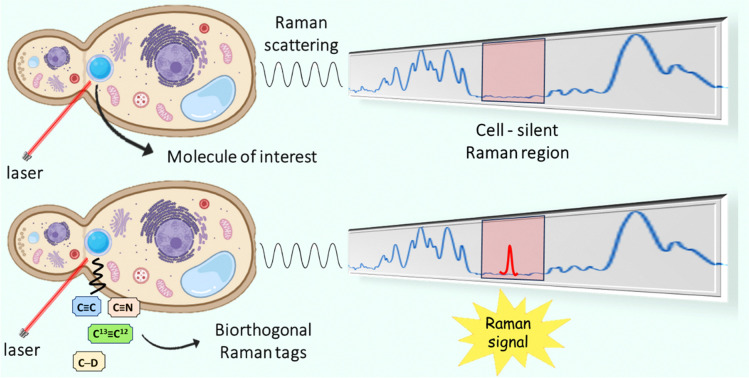

## 1. Introduction

Imaging of molecules in cells and tissues is a fast-growing research field. The current approaches include a range of techniques, of which only a few are of non-invasive nature. Raman spectroscopy is one of them as it is able to reveal molecular bond vibrations in samples of interest through the analysis of the inelastic (Raman scattered) light resulting from its interaction with the sample. Those sample-specific molecular fingerprints which can be acquired in a non-invasive way, led to the employment of Raman spectroscopy in different fields such as pharmaceutics,^1^ regenerative medicine,^2^ pathogen identification,^3,4^ environment and food analysis,^5–7^ art,^8^ forensics,^9^ body fluid analysis,^10–13^ cell therapies,^14,15^ cancer screening^16^ and other disease diagnosis.^17–20^

However, the application of spontaneous Raman spectroscopy on biological samples suffers from (a) inherently weak signal-to-noise ratio and (b) low concentration of the analytes of interest. Over the last decade, the employment of tags has facilitated molecular imaging by improving the sensitivity and chemical contrast of the techniques. Fluorescent tags are commonly used for such purposes, however those are large molecules which entail the risk of disrupting the activity of targeted biomolecules when used for imaging.^21^ On the other hand, Raman tags can be much smaller in size, detecting molecular vibrations of biomolecules with high specificity and minimal interference.

**Fig. 1:**
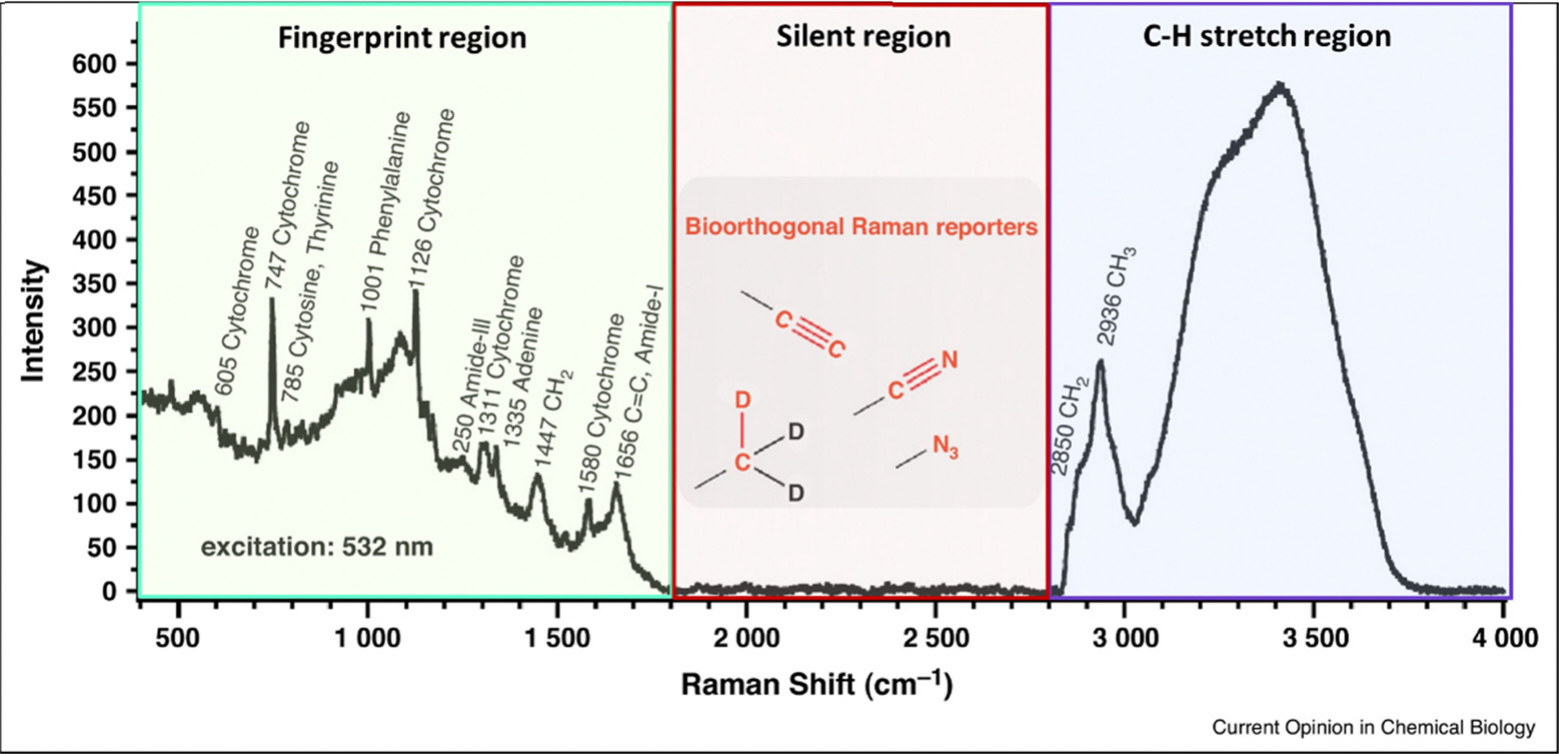
Raman spectrum taken on a live HeLa cell. The cell – silent region (1800–2800 cm^−1^) where bioorthogonal Raman tags exhibit Raman signal is highlighted in the spectrum. Reprinted (adapted) with permission from ref. 25. Copyright 2015 Elsevier.

A recently emerging and highly promising type of Raman tags are those which allow for discrimination against cellular background signal, achieving in this way bioorthogonal Raman imaging. Those tags exhibit a Raman signal at the cell – silent region of the Raman window, commonly defined as the spectral range lying between the fingerprint and high wavenumber regions (1800–2800 cm^−1^). The Raman silent region in cells and biological samples is featureless and characterized by the complete absence of biological molecules’ contribution (Fig. 1). This is because bonds whose vibrations typically exhibit Raman signal in this range (*i.e.* triple bonds), are not present in biological molecules.^22,23^ By employing Raman tags with a distinct signal in the cell – silent region,^24^ one manages to avoid overlapping peaks in the fingerprint window which typically rise from the intracellular species. This allows for improved sensitivity and imaging quality of samples which inherently exhibit weak Raman signal, such as cellular environments and complex biological systems.

Although there is a number of noteworthy review articles in literature discussing the application of bioorthogonal Raman tags, those usually focus on a specific Raman modality^26,27^ or category of tags.^28,29^ In this work, rather than performing an exhaustive overview of Raman tagging for imaging, we specifically attempt to outline the potential of the cell-silent Raman window, focusing on: (a) the approaches for molecular labelling in the cell – silent Raman region and (b) the range of biological applications enabled by the employment of those labels. We eventually hope that this review article will inform researchers on the current applications and potential of tags in the cell – silent Raman window.

## 2. Molecular labelling at the cell – silent Raman window and applications

A number of molecular labels with signals at the Raman cell – silent region which does not interfere with those of natural biomolecules in cells, have been employed from various research groups to enhance detectability. Those tags allow for Raman imaging with minimum interference as they are spectroscopically bioorthogonal and can be either small molecules, polymers or isotope analogues.

### 2.1. Small molecules

The small molecules which are the most widely employed Raman tags in the cell – silent Raman region are low molecular weight molecules, usually containing at least one alkyne or nitrile group (Table S1, ESI†). Those tags provide advantages in terms of small size, photostability and bioorthogonality as they do not react towards endogenous cell biomolecules.

Small molecules have been employed for imaging using three main Raman modalities: (a) spontaneous Raman spectroscopy, (b) stimulated Raman spectroscopy (SRS) and (c) surface enhanced Raman spectroscopy (SERS). Spontaneous Raman spectroscopy is the standard Raman modality which comes with the caveat of weak scattering intensities and therefore long measurement times to acquire signal-to-noise ratios of sufficient quality. SRS and SERS are Raman modalities of higher sensitivity. Unlike spontaneous Raman spectroscopy, SRS uses two laser beams on the sample which induce signal amplification when their frequency difference matches the frequency of a molecular vibration. Similarly to coherent anti-Stokes Raman scattering (CARS), SRS allows for rapid acquisition and provides much higher signal intensities and signal-to-noise ratios compared to conventional RS. However, it also allows for a very limited spectral window (only a few Raman shifts) to be probed at a time. SERS provides a significant (at least 10-fold) local enhancement of the Raman signal typically during contact of the analyte with a metal surface. The downside of the approach is the lack of reproducibility in the SERS signal as it is affected by numerous parameters such as the chemical structure, conformation and orientation of the molecules, saturation of metal surfaces, and varying intensities of the local electromagnetic fields.^14^

#### 2.1.1. Spontaneous Raman spectroscopy

**Fig. 2:**
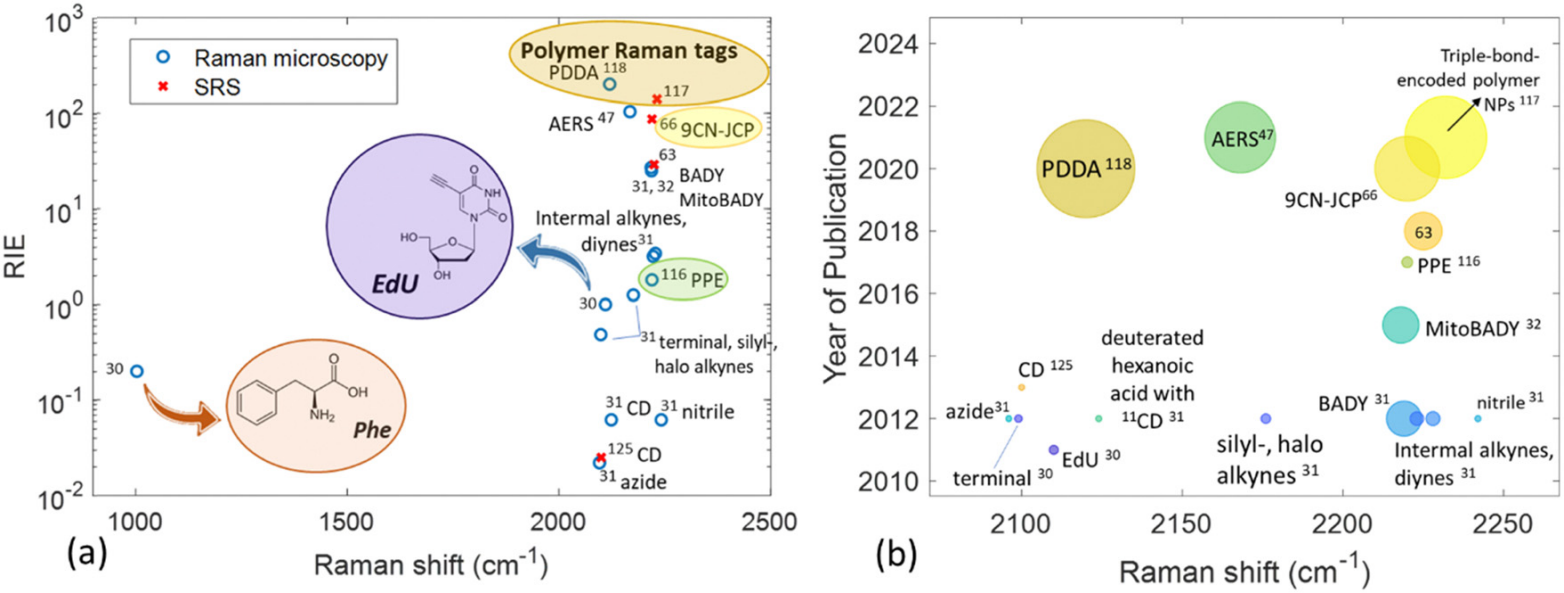
Raman shift of various moieties exhibiting a Raman signal in the cell-silent Raman region as a function of (a) Raman intensity *versus* EdU (RIE) and (b) year of publication. In panel (a) phenylalanine is included for purposes of comparison. In panel (b) the size of the bubble is proportional to the RIE of the tag. The values derived from the literature can be found in Table S2 (ESI†).

One of the earliest employed alkyne-containing small molecules for live cell imaging using spontaneous Raman spectroscopy is EdU (5-ethynyl-2′-deoxyuridine),^30^ an analogue of thymine which covalently incorporates into the DNA and therefore allows for nucleus localization. As EdU Raman signal has been employed across live-cell Raman studies over time, it is now widely used as a standard to assess the relative Raman intensity of different tags (RIE) (Fig. 2).

EdU consists the basis of the alkyne-tag Raman imaging (ATRI) approach which has been established by Yamakoshi *et al.* in 2012.^31^ The same research group explored the position of a variety of alkynes with different structures in order to determine the best tag for cell biomolecules. The alkyne structures have been later assessed *in vitro* through mitochondrial coenzyme Q (CoQ) tagging, demonstrating the imaging feasibility of hydrophobic molecules in live cells by means of spontaneous Raman microscopy. Conjugated diynes exhibit an approximately 5 times higher intensity compared to simple alkynes. The conjugation of an alkyne to an aromatic ring or a second alkyne was shown to exhibit exceptionally strong Raman signal in the cell – silent region. In the case of the ring, the alkyne intensity can be influenced by different type and position of other substituents (*e.g.* phenyl and carbonyl groups) in the ring. The functional groups of nitrile, azide and deuterium have generally shown weaker signal compared to the alkyne tags. As the two alkynes (diynes) conjugated to an aromatic ring yielded a very high Raman intensity (around 25 times that of EdU) the structure has been employed in the form of bisarylbutadiyne (BADY) for mitochondria imaging in live HeLa cells through tracking of the 2220 cm^−1^ Raman band at the cell – silent region.^32^ A multiplex approach using both EdU and BADY has been adopted by Matuszyk *et al*. to explore endothelial dysfunction in *in vitro* models through detection of lipid-rich organelle in endothelial cells.^33^ In other cases, diynes have been conjugated with sphingomyelin, a cell membrane lipid,^34^ and employed as a Raman label in lipid raft measurements monitoring the characteristic 2263 cm^−1^ Raman band.^35^ Jamieson *et al.* employed alkyne tagged fatty acids to monitor their uptake and distribution in single cells using conventional Raman microscopy.^36^ Gala de Pablo *et al*. combined alkyne groups and fluorescence in a single tag in order to image the molecule's distribution in colorectal cancer cells.^37^ Meister *et al*. were the first to employ stable, water-soluble metal-carbonyl (manganese-based) compounds and image their uptake and intracellular distribution in living cancer cells using Raman microscopy.^38^

##### 2.1.1.1. Ratiometric probes

The functional group of nitrile (–C≡N) has been also used as a bioorthogonal tag in cell studies. Yamakoshi *et al.* presented the first structure-based imaging of different forms of a bioactive small molecule (FCCP-carbonylcyanide *p*-trifluoromethoxyphenylhydrazone) in live cells by means of spontaneous Raman microscopy.^39^ The group exploited the nitrile group as an intrinsic Raman tag of FCCP in the cell – silent region to simultaneously visualize the distribution of the two distinct molecular structures – protonated and deprotonated – of the molecule. Boron cluster molecules alone^40^ or conjugated with biomolecules^41^ have been also employed as bioorthogonal tags taking advantage of the B–H stretching frequency band at 2570 cm^−1^ to image their accumulation and uptake in living cells by spontaneous Raman spectroscopy. Another ratiometric Raman sensor of interest was developed by Egoshi *et al.* and evaluate real-time H/D exchange of terminal alkynes in living HeLa cells,^42^ highlighting the utility of non-conjugated d-alkyne tags in neutral or acidic conditions where they can serve as Raman tags to observe similar small molecules in cells. In a more recent development, Yamakoshi *et al*. developed a sensor to monitor the reversible thia-Michael reaction (between an α-cyanoacrylic acid derivative (ThioRas) and thiols) in cellular conditions.^43^ The sensor was able to detect thiol adducts and ThioRas simultaneously in various subcellular locations of HeLa cells. Ratiometric sensors for biologically relevant ion species is another type of sensors which have been employed in the field of spontaneous RS. Tipping *et al.* have developed a novel sensor in a simple paper-based assay format for the detection of fluoride anions using a portable Raman spectrometer,^44^ whereas Takemura *et al*. designed a ratiometric sensor for the detection of Zn^2+^ in live cells using a chelation-based Raman probe.^45^

##### 2.1.1.2. Drug imaging

Intracellular drug localization in cancer cells has been achieved with conventional Raman microscopy in the case of neratinib, a tyrosine kinase inhibitor containing a nitrile group, establishing a novel way of studying drug pharmacokinetics.^46^ A range of azo-enhanced RS molecules (AERS) were employed by Zhang *et al*. for multicolor spontaneous Raman imaging of live cells,^47^ paving the way to visualization of various entities in complex systems.

#### 2.1.2. Stimulated Raman spectroscopy (SRS)

The alkyne-tags have been particularly exploited in the field of stimulated Raman spectroscopy (SRS), one of the nonlinear modalities of RS, providing much stronger signal in a shorter time compared to spontaneous Raman spectroscopy. Alkynes are widely employed as nonlinear vibrational tags through incorporating alkyne-containing small molecules into different cellular macromolecules to achieve respective imaging. By 2012, the well-established case of EdU had been already employed for spontaneous RS imaging of the cell nucleus as it metabolically incorporates to replicating DNA.^30^ It should be noted here that although a popular choice, EdU is linked to toxicity and cycle arrest of cells under long exposure.^48,49^ Hence the alternative of the less toxic alkyne-containing thymidine (2′*S*)-2′-deoxy-2′-fluoro-5-ethynyluridine (F-araEdU), first reported by Neef *et al*.,^50^ is suggested.^51^

**Fig. 3:**
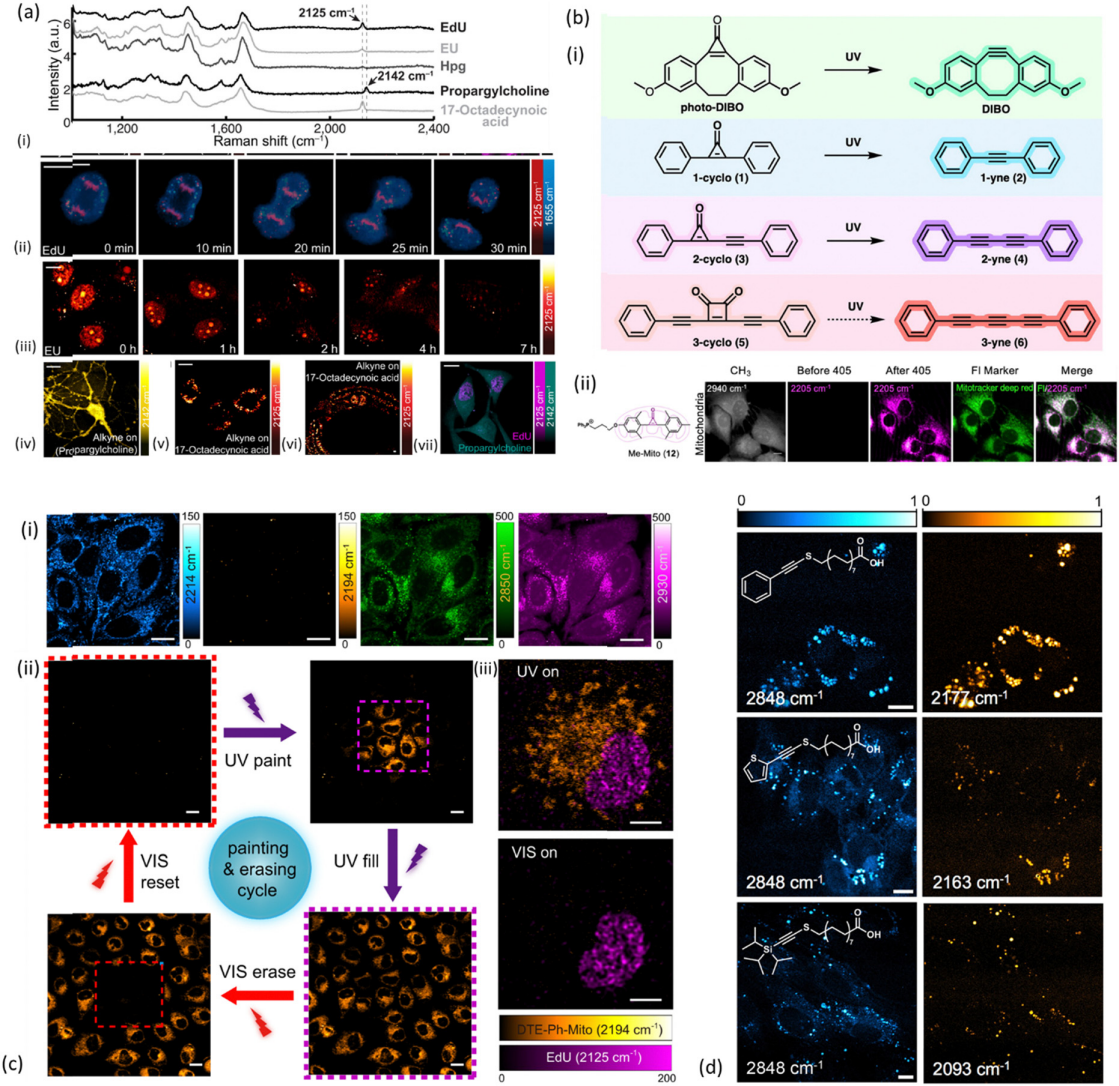
Examples of alkyne-tag SRS imaging applications. (a) Live SRS imaging of *de novo* synthesis of DNA, RNA, proteomes, phospholipids and triglycerides by metabolic incorporation of alkyne-tagged small precursors. (i) Raman spectra of cells incubated with EdU, EU, Hpg, propargylcholine and 17-octadecynoic acid (17-ODYA). (ii) Timelapse imaging of a dividing cell incubated with EdU (100 μM). (iii) Pulse-chase imaging of RNA turnover in cells incubated with 2 mM EU for 12 h and then changed to regular media. (iv) 2142 cm^−1^ image of live neurons incubated with 1 mM propargylcholine (alkyne-on). (v) 2125 cm^−1^ image of live macrophages incubated with 400 μM 17-ODYA (alkyne-on). (vi) 2125 cm^−1^ image of *C. elegans* fed with 17-ODYA (alkyne-on). (vii) Dual-color SRS images of simultaneous EdU (2125 cm^−1^, magenta) and propargylcholine (2142 cm^−1^, green) incorporation. Reprinted by permission from Springer Nature Customer Service Centre GmbH: Springer, Nature Methods, ref. 52, Copyright©2014. (b) (i) Structures and reactions for the photoactivatable generation of alkynes developed by Du and Wei including photo-DIBO, 1-cyclo (1), 2-cyclo (3), and 3-cyclo (5), that could potentially undergo UV-activated alkyne generation. (ii) Structure and image of methylated cyclopropenone probe for photoactivated SRS imaging of mitochondria (Me-Mito, 12) in live HeLa cells together with fluorescence imaging with costained commercial marker used to validate the labeling specificity of SRS probes *via* colocalization. Scale bar: 10 μm. Reprinted (adapted) with permission from ref. 53. Copyright 2022 American Chemical Society. (c) (i) DTE-Ph-Mito labeled live HeLa cells imaged at different Raman frequencies: alkyne stretches of the open-(2214 cm^−1^) and closed-ring (2194 cm^−1^) isomers, CH_2_ symmetric stretch (2850 cm^−1^) and CH_3_ symmetric stretch (2930 cm^−1^). (ii) The cell painting and erasing cycle, through the processes of painting a subset of the cells, lighting (filling) up the whole FOV, erasing a subset of the cells and resetting the whole FOV to the “off” state, using controlled UV/visible irradiations in the purple/red dashed squares. (iii) Dual-color SRS imaging of DTE-Ph-Mito (mitochondria targeting) and EdU (nucleus targeting) co-cultured cells under different irradiation conditions. Scale bars: 5 μm in (iii), 10 μm in (i), and 20 μm in (ii). Reprinted by permission from Springer Nature Customer Service Centre GmbH: Springer, Nature Communications, ref. 54, Copyright©2021. (d) SRS images of NIH3T3 cells incubated with select palmitic acid probes Ph-Alk-S (top), TP-Alk-S (middle), and TIPS-Alk-S (bottom). Left column shows the SRS images taken at 2848 cm^−1^, whereas the right column shows images taken at the maximum of the alkynyl stretching mode (bar = 10 μm). Reprinted (adapted) with permission from ref. 55. Copyright 2023 American Chemical Society.

In 2014, scientists started exploiting the speed and efficiency of SRS technology, with a few studies to be reporting on imaging of the characteristic signal of the alkyne in EdU (2120 cm^−1^) in nucleus of HeLa cells.^51,52^ Wei *et al.* have even demonstrated feasibility of tracking cell division every 5 min during mitosis using EdU (Fig. 3a(ii)), showing that SRS approach is fully compatible with live cell dynamics.^52^ The detection threshold of intracellular EdU was determined at 200 μM.

##### 2.1.2.1. Imaging of macromolecules and metabolites

In the same study, alkyne-tagged small precursors were also metabolically incorporated in RNA, proteomes, phospholipids and triglyceridedes in order to SRS-image the *de novo* synthesis of the biomolecules. More specifically, the alkyne-tagged uridine analog 5-ethynyl uridine (EU) was employed to image RNA turnover as well as its distribution inside HeLa cells (Fig. 3a(iii)), with higher abundance to be found in the nucleoli as expected. An alkyne-tagged analog of methionine (l-homopropargylglycine, Hpg) was employed to visualize newly synthesized proteomes (Fig. 3a(iv)), whereas lipid droplets of THP-1 macrophages were visualized utilizing 17-octadecynoic acid (17-ODYA) as a fatty acid mimic, and its respective band at 2125 cm^−1^ for SRS monitoring (Fig. 3a(v)).^52^ Around the same time, Hong and coauthors were also exploring SRS imaging of alkyne-modified lipids, nucleic acids, proteins and glycans in living HeLa cells. In order to image proteins and fatty acids with high molecular specificity, cells were treated with Hpg and palmitic acid functionalized with an alkyne (17-octadiynoic acid, Alk-16) respectively. In the same way, glycans were imaged in K20 cells treated with peracetylated *N*-(4-pentynoyl)mannosamine (Ac_4_ManNAl), a molecule which can be metabolically converted to the corresponding sialic acid (SiaNAl) and incorporated into the sialylated glycans (2120 cm^−1^).^51^

Apart from macromolecular imaging, the combination of bioorthogonal tags and SRS technology, has been used to explore glucose metabolism. Hu *et al.* have reported monitoring of glucose uptake activity in healthy and cancer live cells as well as *ex vivo* mouse brain tissue using 3-*O*-propargyl-d-glucose (3-OPG), a glucose analogue containing alkyne, which exhibits a signal in the cell – silent Raman region (2129 cm^−1^).^56^ The alkyne in 3-OPG has been later isotopically edited with ^13^C (shifting the frequency from 2129 cm^−1^ to 2053 cm^−1^, ^13^C≡^13^C stretching) and combined with deuterium-labeled glucose (d7-glucose) to consist the basis for a novel two-colour SRS imaging probe to study glucose metabolism in several cell lines and also live mouse brain tissues *ex vivo*. This study achieved to simultaneously image glucose uptake and incorporation activity by combining two different SRS probes with little cross-talk in the cell – silent Raman window, into one.^57^

Boron clusters have been used for direct imaging of cellular uptake in HeLa cells^58^ with the potential to evaluate the distribution of boron drugs in cells and therefore their efficacy during boron neutron capture therapy. A very recent development in the SRS field is the employment of alkyne-based Raman tags with a sulfur-linker, which not only provides an 8-fold enhancement of the alkyne stretching cross-section but also retains a narrow line width of the band.^55^ The suggested probes were shown to be well-tolerated by HeLa cells (Fig. 3d) and metabolically esterified to neutral lipids after cellular uptake.

##### 2.1.2.2. Multiplexed imaging

In terms of multiplexing SRS imaging using bioorthogonal tags, Hu *et al.* have synthesized a series of 20 polyynes with distinct Raman frequencies which were later functionalized into imaging probes and employed for multitarget SRS imaging (CARBOW) of organelles in living cells.^59^ The phenyl-capped polyynes were shown to increase linearly in Raman shift and exponentially in intensity with conjugation length as the number of triple bonds increases from two to six. In a further step, the authors infused polystyrene beads with polyyne mixtures which could be then functionalized with antibodies or enzymes for the purposes of medical diagnostics or drug discovery rather than imaging. Previous attempts of the group include the employment of a narrower palette consisted of 9-cyanopyronin-based dyes (Manhattan Raman scattering – MARS) to image molecules within live neuronal cells and reveal cell-type dependent DNA and protein metabolism using electronic pre-resonance (EPR) SRS.^60^ A year later the same group has expanded the MARS library through ring expansion of the xanthene core, replacement of the core atoms and isotopic substitution, demonstrating enhanced multiplexing capabilities.^61^ All the aforementioned tags were combined with fluorescent probes which are otherwise limited in the ability to resolve colours due to the inherently featureless nature of fluorescence. Chen *et al*. have developed a multiplexed live-cell Raman profiling platform consisting of 14 Raman probes used for the simultaneous evaluation of multiple parameters in the cells.^62^ The platform, which was compatible with live-cell cytometry, consisted of ultra-bright Raman dots (Rdots) conjugated with antibodies and aptamers and was used to analyze the quantification of cell surface protein expression levels, endocytosis activities, and metabolic dynamics of individual live cells.

Later, Wang *et al*. have successfully incorporated Raman tags within a single unnatural amino acid in genetically targeted proteins in HeLa cells to image the alkyne (2135 cm^−1^) carried in the proteins by means of hyperspectral SRS imaging.^63^

More recently, Murphy & Tipping *et al*. have demonstrated the potential of metallacarboranes as Raman reporters by employing boron clusters together with a bis-alkyne and deuterated fatty acid to achieve multiplex Raman imaging in live HeLa cells.^64^ Activatable Raman probes, a group of molecules able to alternate between “on” and “off” states, have found a unique place in multiplex imaging applications over the past few years. Du and Wei were the first to identify the first general design of photoactivatable alkyne-based Raman probes (Fig. 3b(i)) for live-cell multiplexed imaging and tracking.^53^ They spectroscopically investigated a group of cyclopropenones and found that their initially weak Raman signal turned into an intense characteristic alkyne peak at 2171 cm^−1^ after UV irradiation. The photoconversion of cyclopropenones to alkynes consisted the basis for three-color photoactivatable imaging of multiple organelles such as mitochondria (Fig. 3b(ii)), lysosomes, lipid droplets, and endoplasmic reticulum (ER) in live HeLa cells, and demonstrated multiplex subcellular and single-cell tracking. To enhance the detection sensitivity of photoswitchable Raman probes, Wei *et al.* have extended their employment to electronic pre-resonance SRS (epr-SRS).^65^ Electronic pre-resonance SRS has been also employed for the first demonstration of simultaneous imaging of four different enzymes (three aminopeptidases and a glycosidase) in live cells.^66^ The Raman probes targeting those enzymes were synthesized from a scaffold dye of xanthene derivative bearing a nitrile group at position 9 (9CN-JCP), and tuned to different vibrational frequencies by isotope editing of the nitrile group. The advantage of those enzyme-activatable Raman probes is that they are only Raman active in the cell – silent region upon reaction with the enzymes of interest under physiological conditions. More recently, Ao *et al.* developed conjugated diarylethene alkynes yielding “on” and “off” SRS images when irradiated with UV or visible light due to reversible shift of the alkyne group upon photoisomerization.^54^ This property was employed by the authors to achieve painting/erasing of cells with labelled alkyne-diarylethene (Fig. 3c) as well as photoswitchable SRS imaging in HeLa cells through appropriate labelling of mitochondria with the switchable Raman probes.

##### 2.1.2.3. Ratiometric probes

A number of ratiometric probes exploiting Raman signal in the cell-silent window have been developed for bioorthogonal SRS imaging. Wilson *et al.* designed and developed “Mitokyne 1”, the first small molecule sensor for mitochondrial pH monitoring.^67^ Mitokyne 1 was not only able to report subtle pH changes in response to changes in the cell environment, but also monitor mitochondrial dynamics over time in subcellular spatial resolution during mitophagy. In a more recent study, Braddick *et al*. have demonstrated the first ratiometric sensor for carboxylesterase activity.^68^ The sensor was able to determine the intracellular esterase activity in hepatocyte cells based on a bisarylbutadiyne scaffold in a highly sensitive and specific way. Other groups have manufactured alkyne-based ratiometric probes for recording H_2_S level changes^69^ and hydrogen–deuterium exchange (HDX) of terminal alkynes^70^ targeting mitochondria and DNA in living cells respectively. In the latter case, the alkyne-HDX sensor was shown to be highly sensitive to various DNA structures and able to detect UV-induced DNA structural changes.

##### 2.1.2.4. Drug imaging

Another wide field of research taking advantage of bioorthogonal Raman imaging is monitoring drugs whose structure exhibits a signal in the cell – silent window, either inherently or following chemical modification. The advantage of SRS over the spontaneous Raman approach allows for improved detection in clinically relevant drug concentrations. Indeed, SRS has allowed imaging of the alkyne stretch within ponatinib in nano-molar concentrations following treatment of live human chronic myeloid leukemia cell lines.^71^ In a different study Crawford *et al.* used SRS to visualize rhabduscin, a potent immunosuppressant, at the periphery of bacterial cells due to the isonitrile functional group which inherently occurs in the molecule.^72^ El-Mashtoly *et al*. have exploited the inherent acetylene (C≡C) stretching of erlotinib, an epidermal growth factor receptor (EGFR) inhibitor for the treatment of non-small cell lung cancer, to image its uptake, distribution, and metabolism in colon cancer cells by spontaneous Raman microscopy.^73^ Gaschler *et al.* have synthesized a diyne ferrostatin analog exhibiting a Raman band at 2262 cm^−1^, in order to SRS-image the distribution of ferrostatins in living cells and found that they accumulate in lysosomes, mitochondria, and the endoplasmic reticulum.^74^ Drug delivery pathways have been studied in depths of a mouse ear tissue, by probing the terbinafine hydrochloride (TH), an alkyne-bearing antifungal skin drug. The SRS images acquired at 2230 cm^−1^ (band assigned to the internal TH alkyne) showed that the drug tends to accumulate in the lipid part of the tissue, consistent with its lipophilic nature.^52^ Additionally to drug molecule localization, alkyne tags in the form of oligoyne compounds have been employed to monitor the intracellular pH in prostate cancer cells in response to drug treatment.^75^ SRS has been also combined with fluorescence imaging in multimodal approaches for visualisation of intracellular drug uptake in live cells.^76^

### 2.2 SERS tags

Surface enhanced Raman spectroscopy (SERS) is the microscopy variant where bioorthogonal Raman tags and reporters in the cell – silent Raman region find the most applications. The approach provides a 10^6^–10^14^-fold enhancement of the Raman signal due to the local enhancement of electromagnetic fields arising from localized surface plasmons in nanostructured metals when the molecules are adsorbed onto them.^77^ Those rationally designed surfaces such as silver or gold nanoparticles (NPs), can either be label-free or coated with Raman reporters with the latter to be inherently less specific. Label-free SERS approach has been employed for culturing cells on SERS substrates or metal NPs which are then taken up intracellularly in the form of colloids or through endocytosis.^78^ Both specific and non-specific labels have been widely used in *in vitro* and *in vivo* Raman studies.

**Fig. 4:**
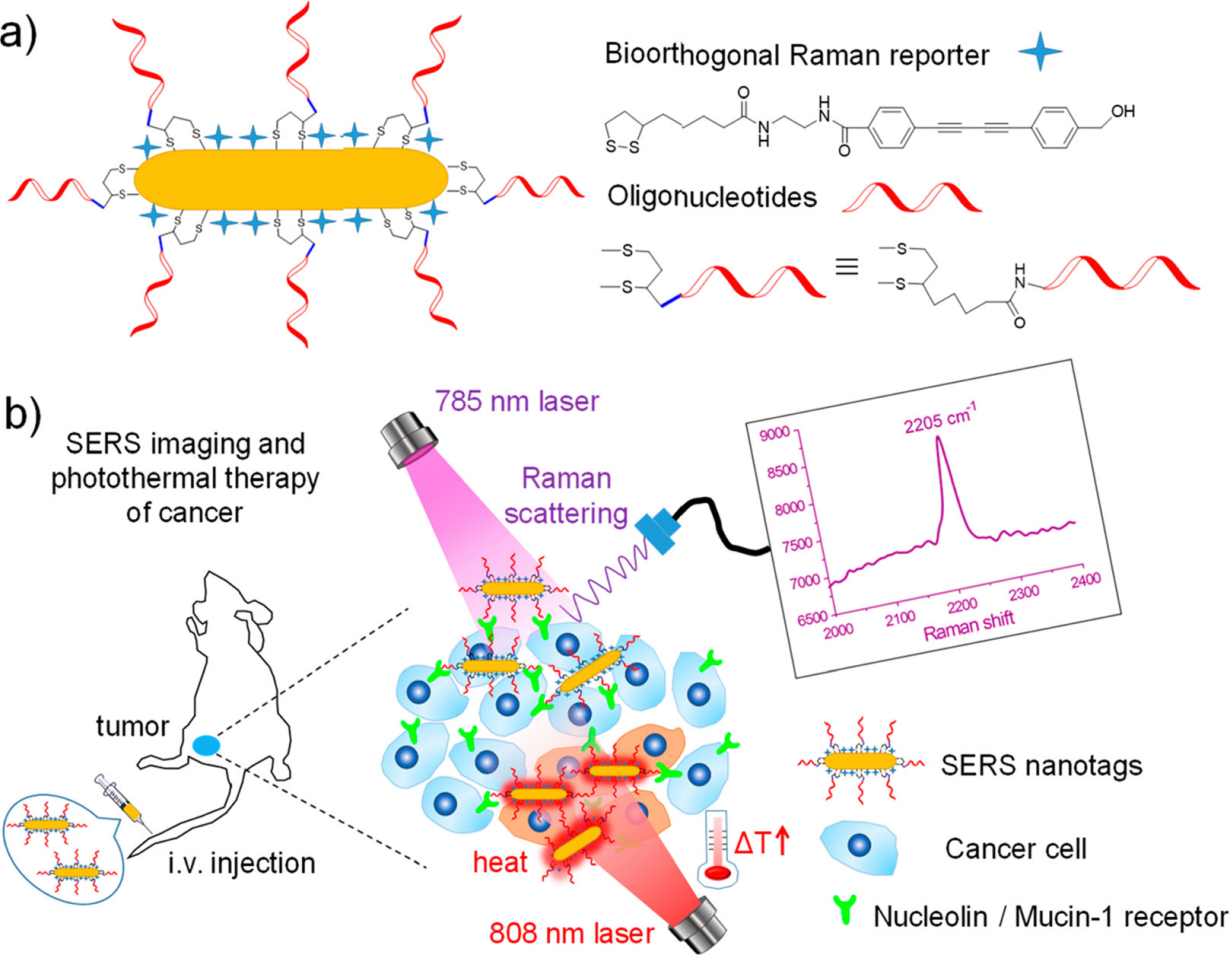
(a) Fabrication of oligonucleotide modified bioorthogonal SERS nanotags. (b) Bioorthogonal SERS nanotags as a precision theranostic platform for cancer detection and photothermal therapy in mice after intravenous (i.v.) injection. Reprinted (adapted) with permission from ref. 82 Copyright©2020, American Chemical Society.

Combining SERS enhancement with the unique contribution of alkyne moieties has led to the development of alkyne-modulated reporter molecules in numerous studies. Yong Chen *et al*. have developed such alkyne-coded SERS tags with a Au@Ag core and conjugated with folate (FA), luteinizing hormone-releasing hormone (LHRH) and CALNNR_8_ peptide, for multiplex Raman imaging on both plant and mammalian cells.^79^ Alkyne groups have been also used as SERS reporters, conjugated to nitroimidazole units, for hypoxia detection, exhibiting a Raman signal around 2200 cm^−1^ in tumours after activation by intracellular reductase under hypoxic conditions.^80^ In a different study, alkyne and nitrile groups have been directly bound to AuNPs *via* σ–π or π–π interactions, to develop SERS tags which following modification with different antibodies, allowed for multiplex imaging of multiple biomarkers expressed in cancer cells and human breast cancer tissues.^81^ From a theranostics point of view, alkyne-bearing aptamer-based SERS gold nanotags (Raman signal at 2205 cm^−1^) were employed by Wang *et al.* to enhance specific recognition of MCF-7 cancer cells in living mice and subsequent targeting using photothermal ablation.^82^ (Fig. 4).

The combination of alkyne tags and silver nanoparticles for SERS detection has been shown to enhance the sensitivity threshold in peptide mixtures down to 100 femtomolar. In a SERS-based approach (alkyne-tag Raman screening – ATRaS), Ando *et al.* combined alkyne moieties with silver nanoparticles to identify binding sites in proteins through peptide screening, specifically the inhibitor-binding site in cysteine protease cathepsin B, a potential drug target for cancer.^83^ Song *et al*. on the other hand exploited silver's large Raman cross-section by protecting it with graphene in order to avoid easy corrosion and enhance NP water solubility.^84^ The fabricated AgCu@graphene nanoparticles were functionalized with alkyne-PEG (Raman reporter) and used to acquire high-resolution Raman images of breast cancer cells at the cell – silent Raman region. On a different note, Plakas *et al.* have modified pyrylium dyes by incorporating alkynes in their chemical structure and altering their functionality in order to produce Raman reporters for the cell – silent region.^85^ The pyrylium-and xanthylium dyes were later adsorbed on gold nanoparticles and identified to be potential candidates for SERS applications at 638 and 785 nm excitation wavelength.

#### 2.2.1. Ratiometric probe sensors

Alkyne-mediated SERS imaging has been widely employed to assess changes in the cellular environment. Qin *et al*. have developed a novel ratiometric SERS nanoprobe based on an alkyne moiety for CO monitoring in living cells and *ex vivo* tissue samples. As the CO in the environment increased, the alkyne ligand was being displaced resulting in the decrease of the alkyne vibrational band (2206 cm^−1^) but also the increase of the metal carbonyl complex signal (∼2100 cm^−1^). The change of the ratio provided a means of measuring carbon dioxide concentration in the sample.^86^ The same group has also developed an alkyne-based SERS nanoprobe for quantifying exogenous and endogenous hydrogen peroxide (H_2_O_2_) in live cells. Similarly to the CO case, in the presence of H_2_O_2_ the alkynyl was released from the surface of the gold nanoparticle, decreasing in this way the Raman signal at 2214 cm^−1^ which was in turn compared to an internally stable alkyne band within the Raman silent window, enabling the ratiometric detection of H_2_O_2_ in the sample.^87^ The same approach was used for the detection of caspase 3 in apoptotic cells and living tissue sections from a rat hepatic ischemia reperfusion (HIR) model,^88^ the monitoring of intracellular endonuclease levels in living cells,^89^ and imaging of hypoxia in living cells and liver tissue from healthy and ischemic rats.^90^ The latter was based on probing of azoalkynes, which were gradually removed from the surface of nanostructures under hypoxic conditions, resulting in the decrease of 2207 cm^−1^ Raman band in the cell – silent window and therefore in hypoxia detection in living cells and tissues.

#### 2.2.2. Metal-carbonyl tags

Apart from alkyne-based tags, metal-carbonyl ones also consist excellent SERS sensors due to the strong σ-and π-acceptor character of CO, which allows it to form strong bonds with metals. Although the first study of organometallic carbonyl compounds in living cells was carried out using spontaneous RS.^38^ further developments took advantage of SRS technology. Kong *et al*. have developed osmium-carbonyl clusters previously employed in infrared studies,^91^ to achieve high contrast live cell imaging (2030 cm^−1^)^92^ as well as monitoring glucose in a human urine environment.^93^ In a later study, they introduced a metal carbonyl fragment (Cr(CO)_3_) to a modified ATP molecule to form a new reporter molecule anchored on a SERS substrate. The Raman signal of the CO stretching vibrations at the cell – silent region (1820 cm^−1^) was shown to exhibit potential in monitoring the pH value of the environment.^94^ A few years later, the metal carbonyl (metal–CO) approach has been adopted, using CO-containing molecules as DNA probes for the SERS detection of cfDNA from Epstein-Barr virus in blood for nasopharyngeal carcinoma, employing the 2113/2025 cm^−1^ ratio.^95^ Last, Panikkanvalappil *et al.* have taken advantage of the enhanced electromagnetic fields in PERS (Plasmonically-enhanced Raman spectroscopy) and combined it with gold nanocubes (PEG/RGD/NLS-functionalized nanoparticles (AuNCs)) to monitor the carbonyl vibration (∼2115 cm^−1^) in the endogenously generated CO and subsequently the dynamics of HO-1 (hemeoxygenase-1) activation in cancerous and healthy live cells undergoing cisplatin treatment.^96^

#### 2.2.3. Nitrile and boron – based tags

The nitrile group has been also used in conjugation with SERS, although not as commonly as the alkyne tags. At a single cell level, Au@4-MB (4-mercaptobenzonitrile) nanoparticles have been used for the detection of cancer biomarkers, probing the 2232 cm^−1^ nitrile vibration in 4-MB.^97^ Yien *et al.* employed a dye which surprisingly acts as a highly sensitive and background-free resonant Raman reporter, Prussian Blue (PB).^98^ Monitoring the 2156 cm^−1^ (CN stretching vibration) Raman band of PB-based SERRS (surface enhanced resonance Raman spectroscopy), they achieved high-sensitivity immunoassay and *in vivo* imaging of cancer cells on a conventional confocal Raman microscope with resonance excitation source.

Water-soluble carborane functionalized silver nanoparticles have been developed and co-functionalized with targeting antibodies by Kennedy *et al.* for tumour cell targeting with anti-EGFR antibodies and delivery of a high concentration of boron using SERS.^99^ It is worth mentioning the additional therapeutic effect of the NPs entailing their ability to produce localized heat leading to damage of the surrounding cellular environment.

#### 2.2.4. Multimodal imaging

Other studies have recruited multiple tags, such as alkynes, azide, nitriles and CD3, with Raman signal at 2121, 2129, 2248 and 2117 cm^−1^ respectively, to perform macromolecular imaging on the cell surface.^100,101^ Combined with a MPBA-AuNP substrate, Lin *et al.* achieved SERS detection of glycans metabolically incorporated with a bioorthogonal Raman reporter on live cells.^101^ Liang *et al.* introduced a novel class of Raman reporter containing carboxylic acid, diynl, and terminal alkyne that could anchor on the surface of gold nanoparticles directly through the C–Au bond formation^102^ acting as a SERS nanoprobe in the biologically silent Raman region. Application of SERS nanoprobes has shown great potential in targeting circulating tumor cells in living mice blood (≈ 2205 cm^−1^) after tail intravenous injection. In human blood, Lin *et al.* combined surface molecularly imprinted polymer technology with gold nanoparticles containing a (C=C) Raman reporter with silent Raman signal at 2024 cm^−1^ and an internal standard (C≡N) for real-time quantitative detection of carcinoembryonic antigen (CEA).^103^

#### 2.2.5. Drug and pesticides applications

For compounds that do not naturally exist in the human body, such as drugs, a number of studies have exploited the combination of SERS and vibrational tagging in the cell – silent Raman window to study and monitor their molecules in biological systems. Koike *et al*. combined 3D SERS imaging and alkyne-tagging for real-time monitoring of small-molecule drug (Alt-AOMK) uptake in live macrophages.^104^ SERS probes in that case were gold nanoparticles introduced into the cell lysosomes by endocytosis. The approach allowed for assessment of the drug uptake with time by tracking the band of 1980 cm^−1^ in the Raman silent window. Tanuma *et al.* combined fluorescence microscopy and SERS with silver nanoparticles to image an alkynylated antidepressant drug (*S*-citalopram-serotonin reuptake inhibitor) in coronal mouse brain tissue using the 2170 cm^−1^ Raman band. The results were promising as the spatial distribution in the tissue as well as the brain transitivity and the serotonin uptake inhibition of the modified drug was not significantly different compared to that of the parent drug.^105^

Pesticides is another category of chemical compounds whose vibrations show up in the cell – silent Raman window. The accumulation of pesticides bearing a CN triple bond (2175 cm^−1^) has been monitored in soybean leaves after incubation of the leaves with SERS nanoparticles.^106^ The same CN stretching mode in cymoxanil but blue-shifted (2130 cm^−1^) due to UV exposure, enabled the detection of the fungicide in fruits and vegetables using the SERS approach.^107^ The nitrile group, which is contained in many agrochemicals, has been also probed with SRS imaging to track the deposition and uptake of commercially available fungicides from maize leaves.^108^ As an alternative for agrochemicals not bearing the nitrile group, the authors suggested deuterium labelling by red-shifting the CH_2_ band from 2850 cm^−1^ to 2100 cm^−1^ (CD_2_ band) in the cell – silent region.

### 2.3. Polymeric nanoparticles

**Fig. 5:**
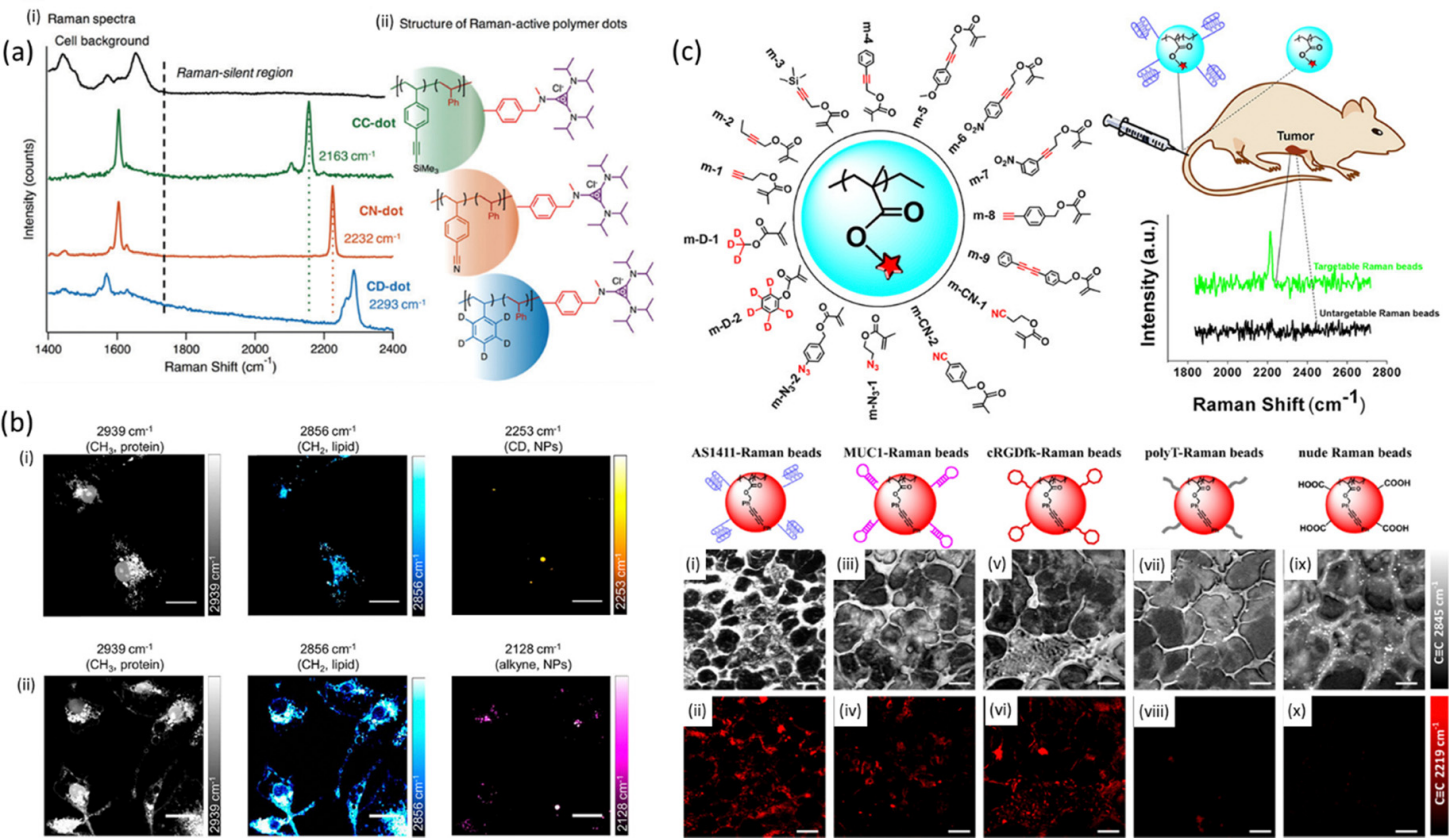
(a) Spontaneous Raman spectra and structure of three distinct Raman-active polymer dots. (i) Spontaneous Raman spectra of nanoparticles with orthogonal vibrational modes of alkyne, nitrile, and carbon–deuterium bonds in the cell Raman-silent region (from 1740 to 2800 cm^−1^). (ii) Schematic structure of three Raman-active polymer dots with the core comprising styrene and styrenic derivatives and the surface coated with trisaminocyclopropenium (TAC) groups. Reproduced from ref. 110 with permission from the Royal Society of Chemistry. (b) SRS imaging of polymer NPs in microglia. Microglia were incubated with PLGA-D NPs (i) or PLGA-alkyne NPs (ii) at 2 × 10^9^ particles mL^−1^ for 24 h before fixing and imaging with SRS microscopy. Scalebars 20 μm. Reprinted (adapted) with permission from ref. 111. Copyright 2019 American Chemical Society. (c) (i) Schematic structure of Raman active monomers which consisted the basis of Raman beads with targeting moieties and employed for tumor targeting in living mice after tail intravenous injection. (ii) SRS images of sectioned slices of tumor tissues from mice intraveneously injected with the targetable Raman beads (AS1411-, MUC1-, and cRGDfk-anchored Raman beads m-9) and untargetable Raman beads (polyT-anchored Raman beads m-9 and nude Raman beads m-9) through the tail vein. SRS images of cell morphology (C–H, 2845 cm^−1^, top row) and different Raman beads (2219 cm^−1^, down row) for AS1411-(a) and (b), MUC1-(iii) and (iv), cRGDfk-(v) and (vi), PolyT-(vii) and (viii), and nude beads (ix) and (x) in tumor tissue slices. Scale bars: 20 μm. Reprinted (adapted) with permission from ref. 112. Copyright 2019 American Chemical Society.

Polymeric nanoparticles are a new class of metal-free Raman probes with a great potential in bioimaging due to lack of cytotoxicity from metal ion leaching, stability and modular chemical functionality.^109^ Min *et al.* have developed polymer dots made of polymer latexes which act as Raman-active nanoparticles for multiplexed live cell imaging.^110^ The Raman dots were free from fluorescent dyes and metals while comprising of a styrene-core and a trisaminocyclopropenium (TAC) surface with different bioorthogonal vibrational tags (alkyne, nitrile, carbon–deuterium bonds) which inherently exhibit signal at the cell – silent Raman window (Fig. 5).

Based on this study, Jin *et al*. have developed organic polymeric nanoparticles as Raman beads with Raman intensity proportional to their size and Raman shift modulated through the attachment of different Raman active monomers such as alkyne, nitrile, azide and carbon-deuterate for multiplex tumor tissue imaging in living mice.^112^ The poly(methacrylate) Raman beads were injected through the tail vein of a mice and fresh tumor tissue was excised and later SRS-imaged at the frequency of alkyne (2219 cm^−1^), paving the way for *in vivo* Raman spectral detection of tumors in mice (Fig. 5c). In the same year, Vanden-Hehir *et al.* incorporated deuterium and alkyne labels into poly(lactic acid-*co*-glycolic acid) (PLGA) synthesizing them into NPs for SRS microscopy imaging. PLGA, a biocompatible and biodegradable FDA-approved polymer, is the mostly used polymer matrix for drug delivery purposes. The authors showed that all synthesized NPs could be imaged within primary rat microglia (Fig. 5b), whereas the alkyne NPs could additionally be visualized in *ex vivo* cortical mouse brain tissue.^111^

**Fig. 6:**
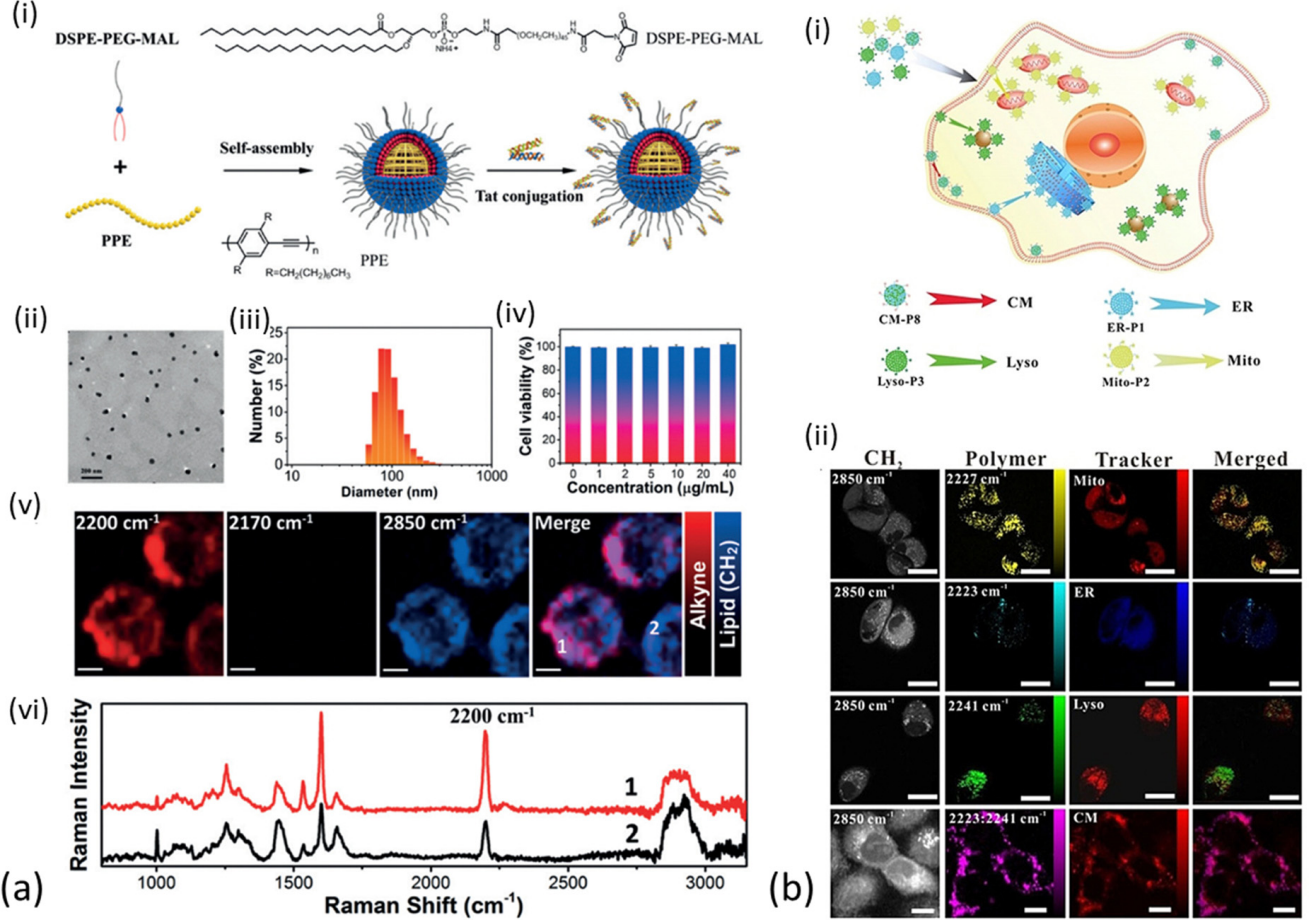
Different applications of polymer nanoparticles on cell imaging (a) (i) schematic illustration for preparation of Tat-PPE NPs. (ii) TEM image of Tat-PPE NPs. (iii) Representative DLS profiles of TatPPE NPs. (iv) Relative viabilities of HeLa cells after incubation with various concentrations of Tat-PPE NPs for 24 h. (v) Living cell Raman images of HeLa cells treated with Tat-PPE NPs (10 mg mL^−1^) for 6 h. Scale bars: 5 mm. (vi) Representative Raman spectra at two locations in living cell as indicated in the Raman image of figure (a) (v). Reprinted by permission from John Wiley and Sons: Angewandte Chemie International Edition, ref. 116, Copyright©2017. (b) (i) Synthesized Raman probes for subcellular organelle-targeted Raman imaging. (ii) SRS imaging of Raman probe entry into live MCF-7 cells incubated with ER-P1, Mito-P2, Lyso-P3, and CM-P8, with MCF-7 cells then co-stained with ER-Tracker Blue-White, MitoTracker Red, Lyso-Tracker Red, and DiD-Tracker Red. Scale bar: 25 mm. Reprinted by permission from John Wiley and Sons: Angewandte Chemie, ref. 117, Copyright©2021.

Conjugated polymer nanoparticles (CPNs) are a recently emerged type of water soluble and highly versatile nano-structured materials with a vast potential in optical imaging and therapy.^113–115^ CPNs exhibit enhanced and distinct Raman signals in the cell – silent Raman region as the alkyne is conjugated into the middle of two aromatic rings, thus the integration of alkyne groups into the long π-conjugated backbone and the high electron-delocalized structure provide great signal enhancement. Shengliang Li *et al.* were the first to employ PPE-based CPNs, manifesting the carbon–carbon triple bond stretching band at around 2200 cm^−1^.^116^ Those were initially assessed spectroscopically and later functionalized with cell penetrating peptides for enhanced live cell Raman imaging. All PPE derivatives exhibited Raman signal in the same area, but varied in strength (up to 1.8 times greater than EdU Raman scattering, Fig. 2). The PPE-based CPNs were shown to be particularly stable in terms of excitation frequency and time and were further modified with Tat peptide to enhance cell uptake of the nanoparticles. Following incubation with HeLa cells, the Tat-PPE NPs were associated with excellent cell viability and heavy distribution in the cell cytoplasm (Fig. 6a). It should be noted here that the PPE-based NPs exhibit important advantages over alkyne-containing small molecules, including enhanced contrast in Raman imaging due to the substantial π-conjugation of the alkyne-containing backbone of conjugated polymer. They are also easy to manufacture and modulate in the lab, although their formulation is usually associated with high costs.

In order to avoid the complex organic synthesis required to design Raman-active polymers with triple-bonds at different positions, Wei Zhu *et al.* have adjusted the ratio between the different monomers for polymerization employing the Raman triple-bond signal ratio to obtain a unique spectral signature of cells.^117^ The triple-bond rich polymer NPs were less complex to synthesize compared to unique triple bond-bearing structures, could enter cells easily due to their small diameter and exhibited stability over irradiation and time and a much stronger Raman signal compared to the EdU standard (between 123–170 times stronger). A number of cell-targeting groups were additionally attached to the polymers for organelle-specific imaging of the endoplasmic reticulum, mitochondria, lysosome and tumour cell membrane in live cells and fixed tissues. In order to verify the targeting efficiency of the Raman probes on the specific sites of the sub-cellular organelles, the cells were co-stained with commercial fluorescent trackers (ER-Tracker Blue-White, MitoTracker Red, Lyso-Tracker Red, and DiD-Tracker Red). After conjugation with targetable moieties (small molecules or specific antibodies), the synthesized polymer NPs were successfully employed as optical probes for multiplex monitoring of tumor microenvironment markers’ expression levels on a single tissue section (Fig. 6(b)).

**Fig. 7:**
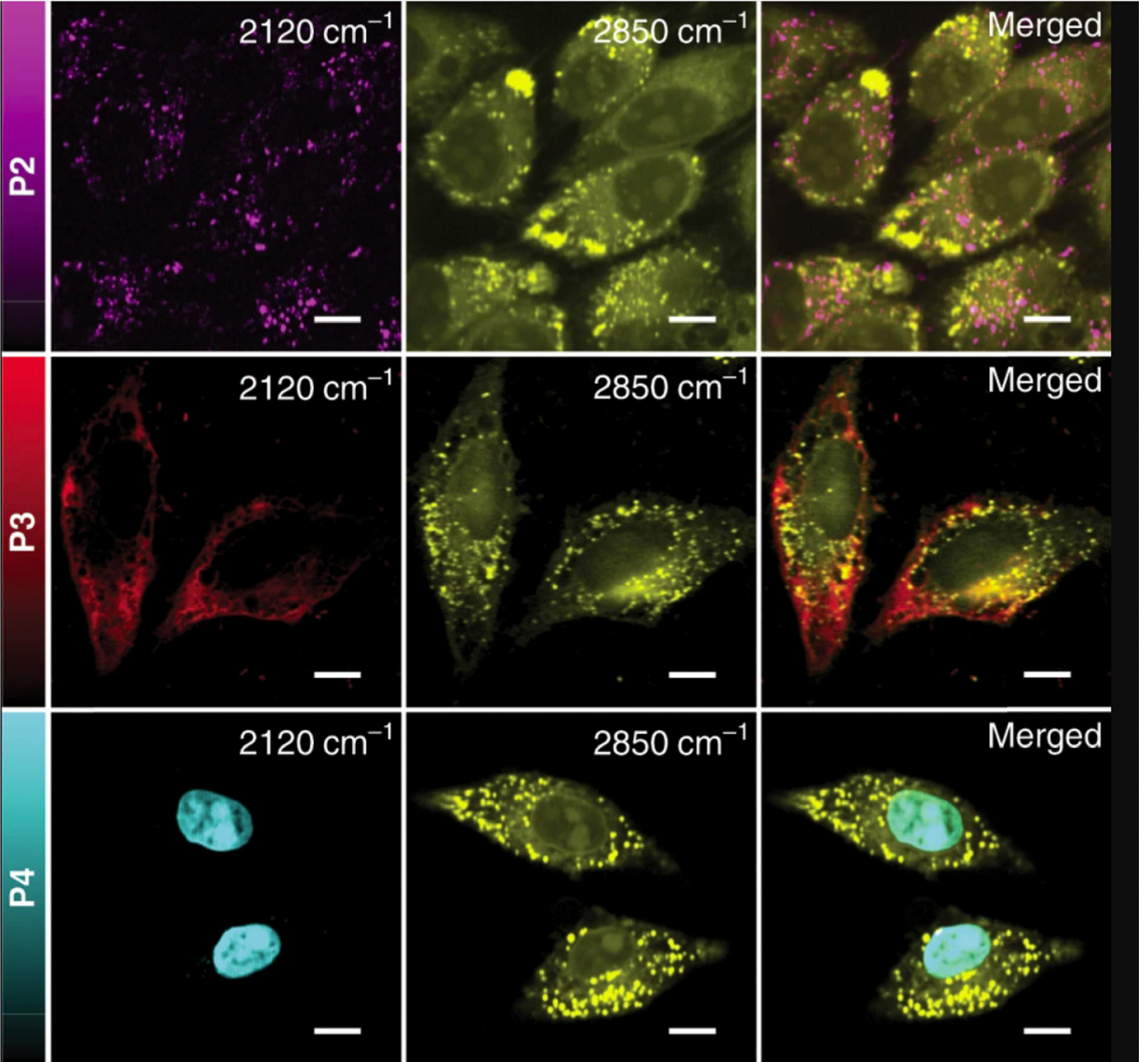
SRS images of HeLa cells treated with 50 μM of P2, P3, and P4, respectively. Images shown from left to right are the alkyne (2120 cm^−1^), lipids (2850 cm^−1^), and merged images. Scale bar: 10 μm. Reprinted by permission from Springer Nature Customer Service Centre GmbH: Springer, Nature Communications, ref. 118, Copyright©2020.

One of the strongest Raman scattering polymer Raman probes (× 10^4^ EdU) in the biologically silent Raman region is based on water-soluble polydiacetylene (PDDA) which was synthesized by Tian *et al*.^118^ The study showed that the PDDA NPs exhibited minimal toxicity in living HeLa cells whereas the propionic acid side chains attached to them allowed for high solubility in water. PDDA can be easily functionalized with Raman vibrational tags enabling targeting of specific subcellular organelles in an SRS modality. The same study employed the 2120 cm^−1^ Raman band in the cell – silent region to visualize the accumulation of different polymer tags in lysosomes, mitochondria and nuclei in HeLa cells (Fig. 7) whereas commercial organelle trackers were used to confirm the specificity of the conjugated probes to the desired targets.

### 2.4. Isotopes

Isotope analogues is a very popular alternative for labelling at the cell – silent Raman region, as they maintain a small size compared to the bulky fluorescent tags. Deuterated analogues are typically safe for patients^119,120^ and provide enhanced signal compared to the parent molecule. The most common isotope labelling for imaging biomolecules due to its stability and lack of radioactivity is deuterium-labelling where the C–H bonds in the molecule are replaced with C–D bonds. Deuterium incorporation leads to a blue shift of the characteristic vibrations typically switching Raman bands from 2800–3000 to 2100–2300 cm^−1^,^121^ enabling in this way imaging at the cell – silent Raman window. However, carbon–deuterium (C–D) Raman tags tend to produce a much weaker Raman signal (about 40 times) compared to the alkyne-tags.^52^

Isotope tags have been extensively used for imaging of various live cell structures using spontaneous Raman spectroscopy. Deuterated derivatives of fatty acids in cytosolic lipid bodies have been employed to image the metabolism and storage of intracellular lipids in phagocytes *in vitro* using Raman microscopy.^122,123^ Van Manen *et al.* have demonstrated for the first time the successful incorporation and detection of isotope-labeled amino acids (tyrosine and methionine) into proteins of single human HeLa cells using their C–D stretching Raman bands in the 2000–2300 cm^−1^ region.^124^ It was further possible to quantify the amino acid incorporation by comparing the labelled to unlabelled Raman bands. A few years later, Wei *et al.* successfully imaged newly synthesized proteins in various live mammalian cells using a combination of deuterium-labeled amino acids and SRS microscopy, an approach clearly superior to spontaneous Raman spectroscopy in terms of speed, efficiency and sample preparation. The study demonstrated potential of protein synthesis and cell dynamics’ visualization through monitoring of the 2100 cm^−1^ Raman band which is assigned to the exogenous carbon–deuterium bonds (C–D).^125^ The same research group has further extended the approach to imaging protein metabolism (synthesis and degradation) in live cancer cells, neurons as well as mouse brain tissue *in vivo* using deuterated amino acids (dAA)^126^ and has later introduced an improved way of dAA administration by infusion *via* the carotid artery in order to minimize the organ bias and enhance the labeling efficiency.^127^ In this study the authors managed to image protein synthesis in different mouse organs such as pancreas, brain and liver, and heterogeneity of protein metabolism in colon tumor tissue, demonstrating the potential of the approach for distinguishing tumors. Recently Spratt *et al.* expanded on their previous work on live cells,^128^ by reporting SRS imaging of deuterated methionine (d_8_-Met) incorporated into Drosophila tissues *in vivo*^129^ and demonstrating in this way the potential of the approach for metabolic imaging of less abundant amino acids in tissue, such as methionine.

Imaging of deuterated lipids has been extensively employed to explore the lipid uptake and metabolic dynamics of d13-palmitic acid^130^ and d38-cholesterol^131^ in macrophages and Y1 adrenal cells respectively, by monitoring the symmetric CD_2_ stretching vibrations between 2100 and 2120 cm^−1^. Villareal *et al.* employed isotopically labeled sterols to SRS-image their distribution in cells infected with Hepatitis C Virus (HCV), demonstrating that one of them (desmosterol) may affect HCV replication *via* a direct mechanism.^132^ Deuterated glucose (d_7_-glucose) and the relevant CD Raman band at 2120 cm^−1^ was recruited to study dynamic metabolism of glucose in single living cancer cells, visualizing *de novo* lipogenesis. An important output of this work was the conclusion that prostate cancer cells exhibit lower levels of *de novo* lipogenesis but higher level of fatty acid uptake compared to pancreatic cancer cells.^133^ Hu *et al.* have also monitored deuterated choline (D_9_-choline, 2188 cm^−1^) in cancer and non-cancer cells using SRS imaging and found that the distribution of choline metabolites inside the nucleus was increased compared to non-cancer cells.^134^ It is worth highlighting that such a conclusion would be otherwise difficult to draw using click-chemistry based fluorescence imaging, due to the low permeability of fluorophores in the nucleus and the loss of small molecules during fixation.

**Fig. 8:**
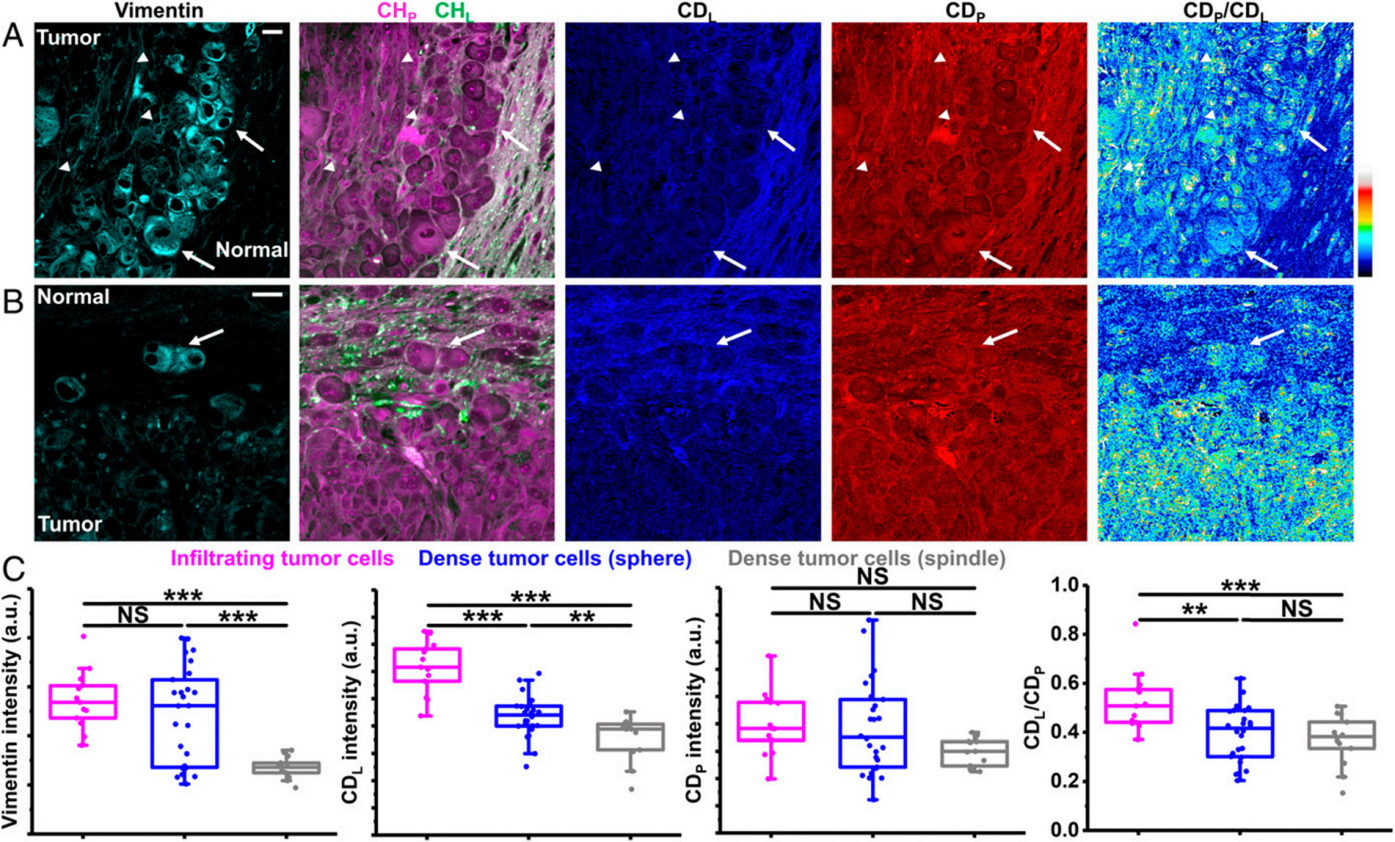
Correlative DO-SRS and immunofluorescence of glioblastoma. (A) Correlative images of immunolabeled vimentin, label-free SRS, and DO-SRS show two types of dense tumor cells: arrows indicate sphere-shaped dense tumor cells and arrowheads indicate spindle-shaped dense tumor cells. (Scale bar, 20 μm.) (B) Correlative images of immunolabeled vimentin, label-free SRS, and DO-SRS show infiltrating tumor cells (arrow). (Scale bar, 20 μm.) (C) Intensities of vimention, CDL, CDP, and CDL/CDP of infiltrating tumor cells (*n* = 13 cells), sphere-shaped dense tumor cells (*n* = 26 cells), and spindle-shaped dense tumor cells (*n* = 13 cells), from one mouse.^138^

Metabolic dynamics of deuterium-labeled saturated and unsaturated fatty acids have been studied in living *C. elegans* using SRS imaging,^135^ revealing that the latter has preferential uptake into lipid storage while saturated fatty acid exhibits toxicity in hepatic cells, and that there is no interaction between the two. Using the same approach, the authors combined SRS with high throughput screening (HTS) to image lipid synthesis in live *C. elegans* fed with deuterated oleic acid, and have subsequently explored protein signaling pathways in lipid metabolism.^136^ Zhang *et al.* used a femtosecond excitation source to increase the SRS signal by 12-fold during imaging of the conversion of deuterated palmitic acid into lipid droplets inside live cells, achieving three-dimensional sectioning of fat storage in live *C. elegans*.^137^ Other advances in the field include the development of a volumetric chemical DO-SRS imaging approach, entailing a Raman-tailored tissue-clearing method using urea and Triton to reduce the refractive index mismatch and achieve a 10-fold depth compared to the standard SRS microscopy. In this way Wei *et al.* observed enhanced lipid synthesis in infiltrating tumors of a mouse xenograft of glioblastoma through monitoring of the deuterium oxide which was metabolically incorporated into the macromolecules (Fig. 8).^138^

Apart from deuterium-labelling, another popular approach for isotopic labelling and subsequent shifting of the C–H vibrations to the cell – silent Raman window is ^13^C substitution. Chen *et al.* have designed three different ^12^C and ^13^C isotopically edited alkynyl metabolic reporters through an alkyne cross metathesis protocol.^139^ The distinct stretching frequencies of the synthesized tags in the cell – silent region were exploited for multiplex chemical imaging of RNA, newly uptaken fatty-acids and DNA synthesis in live mammalian cells. Hu *et al.* have applied both ^12^C and ^13^C labeled EdU isotopologues to monitor DNA synthesis dynamics in mammalian hippocampal slice cultures during traumatic brain injury, observing an upregulation of protein and lipid metabolism following the mechanical injury.^140^

On organism level, Shi *et al.* studied protein synthesis and *de novo* lipogenesis in a variety of cases, ranging from HeLa cells grown in culture medium containing D_2_O to the zebrafish embryos incubated in D_2_O egg water and different organs from mice who drank D_2_O for several days.^141^ Additionally, the group was able to visualize tumor boundaries and intratumoral heterogeneity by monitoring the C–D vibrational modes of deuterated proteins and lipids laying within the 2109–2210 cm^−1^ region. A following study combined deuterated glucose and multichannel SRS imaging (STRIDE-spectral tracing of deuterium) to spectrally trace the Raman signal of deuterium in macromolecules associated with glucose metabolism in both cells and mice. The C–D bonds located at the cell – silent window were used to image glucose metabolism in mice tissues, including tumours, brain, intestine and liver, with high spatial resolution and sensitivity threshold (detection limit of 10 mM of carbon–deuterium bonds).^142^

A different field explored using imaging of the cell – silent Raman region is antibiotic resistance in bacteria. It has been shown that SRS imaging can assess the susceptibility of live bacteria to antibiotics through monitoring of the metabolic uptake of deuterated glucose.^143^ Conventional Raman microscopy on the other hand is able to support a faster antibiotic susceptibility testing (AST) in clinics when coupled with heavy water labeling of bacteria and subsequent monitoring of the C–D vibration in the cell – silent area.^144^

Zhang *et al.* employed isotope labelling in SRS to study cancer metabolism during the epithelial–mesenchymal transition of cancer cells which is a critical step in cancer progression and metastasis. Changes in breast cancer cells have been characterized by tracking glucose metabolism (D7-Glc, 2133 cm^−1^), protein synthesis (CD-AA, 2133 cm^−1^), fatty acid metabolism (d31-PA, 2109 cm^−1^) as well as propargylcholine through its inherent alkyne (2142 cm^−1^).^145^ Shen *et al.* treated HeLa cells with deuterated palmitate (CD_2_ symmetric stretch vibration at 2101 cm^−1^) and SRS-imaged them to shed light on how lipid metabolism modulates endoplasmic reticulum (ER) membrane phase.^146^ The study revealed for the first time that nonequilibrium metabolic activity can lead to separation from the presumably uniformly fluidic ER membrane.

Apart from deuterium-treated cellular macromolecules, deuterated drugs and drug carriers also exhibit a signal in the cell – silent Raman window and can be therefore used to monitor their distribution. Matthaeus *et al.* employed spontaneous Raman microscopy to monitor liposome-to-cell interactions in human breast adenocarcinoma MCF-7 cells where deuterium-labelled liposomal drug carriers were introduced, by tracking the C–D stretches of the aliphatic side chains between 2000 and 2310 cm^−1^.^147^ In the SRS field, *ex vivo* monitoring of topical and transdermal delivery of deuterated ibuprofen (Raman band at 2120 cm^−1^) has been shown feasible on mice ears.^148^ Deuterated active molecules (glycerol, jasmonic acid and heavy water) have been further mapped within the depth of artificial and human skin using single-color coherent anti-Stokes Raman spectroscopy (CARS).^149^ Both the resonant (2100 cm^−1^) and non-resonant (2250 cm^−1^) C–D stretching vibration were employed to construct the normalized CARS signal. Last, although not a drug, dimethyl sulfoxide (DMSO) in its deuterated form has been applied and imaged *in vivo* on the forearm of a volunteer by tuning to the C–D stretching vibration at 2125 cm^−1^.^150^

## 3. Discussion-prospects and potential

The employment of Raman spectroscopically bioorthogonal probes for imaging is constantly gaining ground thanks to recent chemistry innovations. Although alkynes, nitriles and deuterated molecules are not normally present in mammalian cells, they are abundant in plants, pharmaceutical molecules and natural products, extending the applications of the cell – silent Raman imaging to pharmacognosy and medicinal chemistry. This provides a great potential as Raman imaging can take place without further modification of the molecules. Alkyne-bearing components can be found as secondary metabolites in food plants, for example the falcarinol-type polyacetylene compounds exhibit a characteristic signal at 2250 cm^−1^ due to the symmetric stretch of the conjugated triple-bond polyacetylene system.^151^ The alkyne moiety is also inherently found in many newly synthetized biologically active molecules which are of great interest for medicinal chemistry. Examples include molecules of excellent bioavailability, such as free fatty acid receptor 1 (FFA1/GPR40) Agonists^152^ and alkynylphenoxyacetic acid CRTH2 (DP2) receptor antagonists,^153^ and strong therapeutic potential such as synthetic acetylenic lipids^154^ and phenylethynylbenzenesulfonamide regioisomers.^155^

Biomedical applications gain particular benefit from the employment of bioorthogonal RS probes due to the inherently weak signal-to-noise ratio associated with probing biomolecules. In this review article we focus on the different types of Raman tags employed in the cell – silent region (1800–2800 cm^−1^). The bioorthogonal analogues ranging from small molecules to polymer and isotope tags have been employed in various Raman modalities over the past few decades, starting with spontaneous Raman spectroscopy and evolving into variants of higher sensitivity such as SERS and the recently emerged SRS microscopy where the coherent nature of signal generation is exploited. Specifically coupling SRS with bioorthogonal Raman reporters has shown immense potential in biomedical imaging due to high sensitivity and molecular specificity. In depth Raman modalities such as spatially-offset and transmission Raman spectroscopy,^156–160^ merged with surface-enhancement Raman spectroscopy (surface-enhancement spatially offset Raman spectroscopy-SESORS), have yet to be combined with bioorthogonal cell – silent Raman tags. We believe that such an approach would entail great potential given the recent advances in the field of theranostics.^161^

Bioorthogonal vibrational tags are generally superior to fluorescent proteins and organic dyes for imaging of small biomolecules due to their size. As fluorescent tags (1–10 nm in size)^26^ are typically much larger compared to small-molecule Raman tags (starting with a few atoms), they can potentially disrupt the native cellular environment. The least perturbing vibrational tags are the ones formed through stable isotope substitution as they are tiny in size. However, Raman cross-sections of these tags are also small and their Raman signal very weak, making them suitable for imaging of small but abundant biomolecules.^162^ Alkyne moieties on the other hand have much larger Raman cross-sections, yielding a stronger Raman signal, but also introducing structural alterations which could potentially modify the chemical activity of the molecules.^56^

Given the inherently weak nature of Raman scattering, high intensity lasers and long acquisition times are often required to achieve sufficiently high signal quality while at the same time risking cell damage. Therefore the design and employment of Raman tags yielding a strong Raman signal in a suitable spectral window is of major significance for relevant applications. A significant amount of research has been conducted to correlate Raman tag structure with Raman intensity and explore frequency and intensity modulation of Raman tags. Conjugation effects, isotope editing and end-capping variation can impact Raman tags’ properties and have been recently discussed as design strategies.^163^ For example, Raman cross-section of the tags can be influenced by substitution groups in molecules where conjugation effects are induced. In those molecules both Raman intensity and frequency are affected by changes in the spring constant in Hooke's law and the polarizability of the chemical bond which is in turn amplified, leading to enhanced Raman signals.^164^ Further signal enhancement can be achieved through excitation in the electronic pre-resonance window by exciting with a laser wavelength slightly lower in energy than the desired electronic transition.^165^

Although structure–activity relationship of Raman tags has been described in more detail above, we summarize some general considerations on the employment of tags for enhanced Raman sensitivity:

(a) Alkynes conjugated to an aromatic ring exhibit stronger Raman scattering than without the ring. In the first case, the Raman cross-section of the molecule can increase when the π-orbital is extended in the direction of the alkyne stretching due to the relative substituent-conjugated alkyne position on the aromatic ring.

(b) Although the functional groups of nitrile, azide and deuterium exhibit weak Raman intensity (compared to alkyne), their small size can be of benefit for minimum interference in the cell environment compared to aromatic rings. Small alkyne tags are generally considered promising as they are likely to retain the biological activity of the parent molecule while at the same time exhibiting a sufficiently strong Raman signal to be detected.^31^

(c) Introducing diyne in the structure, efficiently increases the Raman scattering cross-section (up to 20 times when the diyne is conjugated to two aromatic rings^166^). Further addition of triple bonds (between 2 and 6) increases the Raman signal exponentially.^59^ Apart from multiple alkynes, a sulfur linker can also enhance the tag signal through n–π conjugation.^55^

(d) Conjugation of an azobenzene moiety has demonstrated enhancement of Raman signal greater than 4 orders of magnitude compared to EdU.^47^

It has been suggested that a diyne would be a suitable sensitive tag for nonaromatic compounds such as lipids. In the case of an initial compound with an aromatic moiety, the introduction of a small ethynyl group at an appropriate position on the aromatic ring could be an excellent choice to generate Raman signal in the cell-silent region.^31^ Although not much has been reported on structure–property relationship of Raman tags, it is expected that this addition would not significantly change the biological properties of the molecule.

In terms of chemical characteristics, the Raman tags should ideally exhibit minimal cytotoxicity and immune response, and high biodegradability which allows for fast decomposition and removal from the living system.^163^ EdU, the widely used bioorthogonal standard for cell imaging which is essentially an alkyne-tagged thymidine analogue, is known to incorporate into the cell DNA and be chemically stable and non-volatile. Although alkyne tags are relatively chemically stable as they do not to react with other molecules in biological systems,^167^ correlation between structure and chemical properties of most Raman tags has not been sufficiently discussed in literature.

PDDA based tags, developed by Luo *et al.*,^118^ exhibit one of the strongest Raman signals in the biologically silent region as well as minimal toxicity on living cells. PDDA has been also reported to disintegrate rapidly and completely decompose within a week when exposed to air and sunlight, yielding the biocompatible succinic acid as a major degradation product.^168^ Self-degradability of conjugated polymers through photooxidation holds great promise for their potential as biocompatible Raman tags. The engineered polyynes developed by Hu *et al.* for multitarget SRS imaging of living cell organelles, were assessed to have good chemical stability under ambient conditions and a high photostability (>98%) during SRS measurements.^59^ Other tags which have been assessed in terms of stability over time in the cellular environment (through NMR assays) include sulfur-linked phenylacetylene tags.^55^

Isotope analogues (typically deuterium and carbon-13) tend to retain the size and electronic characteristics of the parent molecules.^27^ For example, the replacement of hydrogen with deuterium, which is the smallest possible chemical change, increases the weight of the molecule and subsequently the strength and the resistance of the molecular bond to cleavage which in turn renders it more metabolically stable.^120^ However this subtle change does not only affect metabolism but also the toxicity and pharmacokinetic profile of the molecule. Drug research over the past few decades has shown that although deuterated compounds exhibit increased metabolic stability, the efficacy and safety may be compromised.^169^ As a matter of fact, deutetrabenazine, a deuterated form of tetrabenazine, is currently the only drug approved for clinical use.^170^ Deuterated analogues are typically safe to handle and dose to patients, however more research is needed to establish their safety profiles *in vivo*.

Among the various bioorthogonal Raman tags, the polymer ones are of the most promising in terms of high sensitivity and chemical properties. For example, PLGA is the most extensively employed polymer in drug delivery research. Its biocompatible and biodegradable nature makes it very attractive as it breaks down into lactic and glycolic acid, molecules which are later metabolized in the Krebs cycle. As PLGA has already been approved by the Food and Drug Administration (FDA) and the European Medicines Agency for drug delivery purposes in clinics,^171^ the future for other biopolymer nanoparticles which also break down into monomers naturally present in the body looks encouraging.^172,173^

Raman spectroscopy generally suffers from low sensitivity and is unable to detect low concentration molecules (< 100 μM). However, when combined with an appropriate Raman modality, Raman tags can exhibit a further enhanced signal. The combination of SRS technology and bioorthogonal tags currently provides undoubtedly the best imaging capabilities in biomedical systems.^27,174^ As the whole range of the cell – silent Raman spectrum has not been fully exploited yet, we expect a combination of chemistry tag design, computational power and technical instrument advances (*e.g.* recent quantum cascade lasers) to bring a breakthrough in the employment of highly specific and sensitive bioorthogonal Raman tags to biomedicine, clinical diagnosis and eventually health care.

## Author contributions

Conceptualization: M. V., V. G., C. C.; investigation: M. V., K. S. and N. K.; data curation, M. V. and N. K.; writing—original draft preparation, M. V.; writing—review and editing, M. V., V. G., C. C.; supervision: C. C.; resources: V. G., C. C.; funding acquisition: C. C. All authors have read and agreed to the published version of the manuscript.

## Conflicts of interest

There are no conflicts to declare.

## Supplementary Material

CB-005-D3CB00217A-s001
