# Supplementary material for: Biomedical applications, perspectives and tag design concepts in the cell – silent Raman window
Source: RSC Chem Biol. 2024 Feb 12;5(4):273–92. doi: 10.1039/d3cb00217a (PMC10989507; doi:10.1039/d3cb00217a)
Supplement: CB-005-D3CB00217A-s001 [file CB-005-D3CB00217A-s001.pdf]

Electronic Supplementary Material (ESI) for RSC Chemical Biology.
This journal is © The Royal Society of Chemistry 2024

SUPPLEMENTARY

Table SI: Studies of various Raman modalities recruiting bioorthogonal tags and their respective applications.

Authors	Year of publication	Raman mode	Probing	Type of functional group	Application
Yamakoshi et al[30]	2011	Spontaneous RS	EdU- DNA base	alkyne	Imaging of an alkyne-modified molecule (EdU) in living cells.
Yamakoshi et al[31]	2012	Spontaneous RS	coenzyme Q (CoQ)	diyne	Multicolor imaging of small molecules in living cell
Yamakoshi et al[32]	2015	Spontaneous RS	BADY	diyne	Mitochondria imaging in live cells
Matuszyk et al[33]	2021	Spontaneous RS	EdU + MitoBADY, falcarinol	Alkyne, diyne	Multiplex Raman imaging of lipid-reich organelles in endothelial cells
Cui et al[34]	2015	Spontaneous RS	Sphingomyelin	Alkyne, diyne, deuterium	High contrast imaging of lipid rafts
Ando et al[35]	2015	Spontaneous RS	Sphingomyelin	diyne	Imaging of sphingomyelin distribution in lipid monolayer
Jamieson et al[36]	2018	Spontaneous RS	Alkyne tagged fatty acids	alkyne	Monitoring intracellular uptake and localisation of FAs
Gala de Pablo et al[37]	2018	Tandem fluorescence and RS microscopy	DC473 (combining alkyne group and fluorescence)	alkyne	Molecule localization in colorectal cancer cells.
Meister et al[38]	2010	Spontaneous RS	$[Mn(tpm)(CO)_3]Cl$	carbonyl	Imaging of Metal–Carbonyl Complexes in cells
Yamakoshi et al[39]	2014	Spontaneous RS	carbonylcyanide p-trifluoromethoxy-phenylhydrazone (FCCP)	nitrile	Simultaneous imaging of two

					different molecular structures (protonated and deprotonated FCCP)
Tarrés et al[40]	2014	Spontaneous RS	Cobaltabisdicarbollide (COSAN)	Carborane (B–H)	Detection of boron cluster molecules in living cells
Mochizuki et al[41]	2019	Spontaneous RS	Boron clusters	Carborane (B–H)	Molecular targeting in the cell
Egoshi et al[42]	2021	Spontaneous RS	Deuterated terminal alkynes	C–D	Evaluation of H/D exchange of terminal alkynes in living HeLa cells
Yamakoshi et al[43]	2023	Spontaneous RS	Thiol adducts and ThioRas	nitrile	Monitoring of thia-Michael reaction
Tipping et al[44]	2020	Spontaneous RS	4-((trimethylsilyl)ethynyl)benzonitrile	alkyne	Detection of fluoride anions
Takemura et al[45]	2020	Spontaneous RS	dipycolylaminoethyl aniline	acetylene	Detection of Zn^{2+} in live cells
Aljakouch et al[46]	2018	Spontaneous RS	Neratinib (TKI inhibitor)	nitrile	Monitoring drug metabolism in cancer cells.
Tang et al[47]	2021	Spontaneous RS	Azo-enhanced RS molecules	alkyne	Multicolor spontaneous live cell imaging
El- Mashtoly et al[73]	2014	Spontaneous RS	Erlotinib	acetylene	Drug distribution and metabolism
Hong et al[51]	2014	SRS	Homopropargylglycine, EdU, propargylcholine, Sialylated glycans.	Alkyne	Live- cell Imaging of alkyne- tagged biomolecules
Wei et al[52]	2014	SRS	EdU, uridine-, methionine-, fatty acid- analogue, terbinafine hydrochloride	alkyne	Cell division and drug delivery tracking
Du and Wei[53]	2022	SRS	Photoactivatable Raman probes	alkyne	Live-cell multiplexed imaging and tracking
Ao et al[54]	2021	SRS	diarylethene	alkyne	Photoswitchable SRS imaging

Li et al[55]	2023	SRS	Palmitic acid	Alkyne	Imaging and cellular uptake of the tag
Hu et al[56]	2015	SRS	3-OPG	alkyne	Monitoring of glucose uptake activity in live cells and ex vivo mouse brain tissue
Long et al[57]	2018	SRS	D7-glucose, 3-OPG-$^{13}C_3$ probes	^{13}C edited alkyne, deuterium	Glucose metabolism in cell lines and live mouse brain tissues ex vivo
Asai et al[58]	2019	SRS	Disodium mercaptoundecahydrododecaborate (BSH)-cholesterol	Carborane (B–H)	Cellular uptake of boron cluster
Hu et al[59]	2018	SRS	Carbow	polyynes	Mulptiplex imaging of organelles in living cells
Wei et al[60]	2017	epr- SRS	MARS pallete	Alkynes, nitriles	Multiplex optical imaging within live neuronal cells
Miao et al[61]	2021	epr- SRS	Expanded MARS pallete	Alkynes, nitriles	Multiplex optical imaging
Chen et al[62]	2021	SRS	Rdots	Polyynes	Enhanced multiplex live cell profiling
Zhang et al[63]	2018	SRS	Unnatural amino acid	alkyne	Imaging of genetically targeted proteins in HeLa cells
Murphy & Tipping et al[64]	2023	SRS	Boron clusters	Carborane (B–H)	Multiplex optical imaging in live HeLa cells
Fujioka et al[66]	2020	epr- SRS	Enzyme substrates	Isotopically edited nitrile	Simultaneous enzyme imaging
Wilson et al[67]	2021	SRS	Mitokyne	alkyne	Mitochondrial pH monitoring
Braddick et al[68]	2023	SRS	bisarylbutadiyne	alkyne	Carboxylesterase activity monitoring
Zeng et al[69]	2018	SRS	2-yne Mito NH2	alkyne	H_2S imaging in mitochondria

Bi et al[70]	2022	SRS	Alkyne- Hydrogen–Deuterium exchange (HDX) of terminal alkynes	alkyne	Sensing of DNA structural changes
Sepp et al[71]	2020	SRS	Ponatinib	alkyne	Intracellular distribution of Ponatinib in Live Cells
Crawford et al[72]	2012	SRS	Rhabduscin	Isonitrile	Drug localization at the periphery of bacterial cells
Gaschler et al[74]	2018	SRS	Ferrostatin analog	diyne	Distribution of ferrostatins in living cells
Wilson et al[75]	2020	Spontaneous RS and SRS	Oligoyne compounds	alkyne	Intracellular pH sensing.
Tipping et al[76]	2017	SRS	(PhDYANS and BADY-ANS	Diyne	Intracellular drug uptake
Chen et al[79]	2016	SERS	alkyne-coded SERS tags with a Au@Ag core	Alkyne	Multiplex Raman cell imaging
Kurihara et al[80]	2016	SERS	hypoxia-detecting probes	acetylene	Hypoxia detection
Li et al[81]	2019	SERS	AuNPs	alkyne and nitrile groups	Imaging of cancer biomarkers in cells and tissues
Wang et al[82]	2020	SERS	aptamer-based Au nanotags	Alkyne	MCF-7 targeting for photothermal ablation in living mice
Ando et al[83]	2016	SERS	Ag NPs nanoparticles	alkyne	Cancer drug target identification
Song et al[84]	2014	SERS	AgCu@graphene NPs functionalized with alkyne-PEG	Alkyne	High resolution cell Raman imaging
Plakas et al[85]	2022	SERS	Alkyne- modified pyrylium dyes	alkyne	Identification of SERS reporters.
Qin et al[86]	2020	SERS	Porous silica- coated AuAg alloy NPs	alkyne	CO monitoring in cells/ tissues
Si et al[87]	2019	SERS	Porous silica- coated AuAg alloy NPs	alkyne	H_2O_2 detection in live cells
Qin et al[88]	2018	SERS	Au nanorod- based nanoprobe	alkyne	Caspase 3 detection in live cells/ tissues
Si et al[89]	2018	SERS	Alloyed Au/Ag Nanospheres	alkyne	Detection of intracellular

					endonuclease levels in living cells
Qin et al[90]	2019	SERS	Ag/Au alloy NPs	azoalkyne	Hypoxia detection in cells/ animal tissues
Kong et al[92]	2012	SERS	Au NPs	carbonyl	High-contrast live-cell imaging
Kong et al[93]	2013	SERS	Au NPs	carbonyl	Glucose monitoring in urine
Kong et al[94]	2014	SERS	Gold coated planar substrate	carbonyl	pH monitoring
Lin et al[95]	2018	SERS	SERS- active substrate	carbonyl	Epstein-Barr virus cfDNA detection in blood
Panikkanvalappil et al[96]	2019	Plasmonically Enhanced Raman Spectroscopy (PERS)	Gold nanocubes (PEG/RGD/NLS-functionalized NPs)	carbonyl	Monitoring HO-1 activation in live cells during cisplatin treatment
Ma et al[97]	2017	Interference-Free SERS (IF-SERS)	Au@4-MB NPs	nitrile	Cancer biomarkers profiling at single-cell level
Yin et al[98]	2017	SERRS	Au@PB NPs	nitrile	High-sensitivity immunoassay and cancer cell imaging
Kennedy et al[99]	2009	SERS	Carborane-coated silver NPs co-functionalized with anti-EGFR antibodies	Carborane (B–H)	Tumour cell targetting and boron delivery to cancer cells
Lin et al[101]	2013	SERS	MPBA-Au NPs	alkyne	Detection of cell surface glycans
Liang et al[102]	2018	SERS	Gold nanoprobes	alkyne	Tumor Cell targeting in living mice
Lin et al[103]	2019	SERS + SMIP (Surface Molecularly Imprinted Polymer) technology	Gold NPs	acetylene	Detection of cancer biomarker (CEA) in human blood

Koike et al[104]	2020	3D SERS	Gold NPs	alkyne	Real-time monitoring of small- molecule drug uptake in live macrophages
Tanuma et al[105]	2020	Fluorescence microscopy + SERS	Silver NPs	alkyne	Imaging of antidepressant drug analogue in mouse brain
Wu et al[106]	2021	SERS	Au NPs	nitrile	Monitoring of pesticide residues in soybean plants
Mi et al[107]	2021	SERS	Au NPs	Nitrile after UV irradiation	Determination of cymoxanil residue in food
Hu et al[110]	2017	SRS	Polymer dots	Alkyne, nitrile, C–D	Multiplexed live cell imaging
Vanden-Hehir et al[111]	2019	SRS	PLGA NPs	Deuterium and alkyne	Mouse brain tissue imaging
Jin et al[112]	2019	SRS	Raman beads	Alkyne, nitrile, azide, C–D	Supermultiplex barcoding
Li et al[116]	2017	Spontaneous RS	PPE- based CPNs	Alkyne	Enhanced live cell imaging
Zhu et al[117]	2021	SRS	Polymer NPs	Alkyne	Highly sensitive multi-color biomedical imaging
Tian et al[118]	2020	SRS	PDDA NPs	Alkyne	Organelle- targeting live-cell imaging
Matthaus et al[122]	2012	Spontaneous RS	Deuterated fatty acids, deuterated cholesterol	C–D	Imaging of Intracellular Lipid Metabolism in Macrophages
van Manen et al[123]	2005	Raman microscopy	Deuterated arachidonic acid	C–D	Imaging of lipid bodies in leukocytes
van Manen et al[124]	2008	Spontaneous RS	Deuterated tyrosine and methionine	C–D	Imaging of Protein Metabolic Labeling in Single Human Cells

Wei et al[125]	2013	Spontaneous RS	Deuterated amino-acids	C–D	Imaging of newly synthesized proteins in live mammalian cells
Wei et al[126]	2015	Spontaneous RS	Deuterated amino-acids	C–D	Imaging of protein metabolism in live cancer cells, neurons and mouse brain.
Shi et al[127]	2018	Spontaneous RS	Deuterated amino-acids	C–D	In vivo imaging of protein metabolism in mice
Spratt et al[128]	2022	Spontaneous RS	d_8-Met	C–D	Imaging of cellular uptake of deuterated methionine (d_8-Met) in live HeLa cells
Spratt et al[129]	2023	Spontaneous RS	d_8-Met	C–D	Imaging the uptake of deuterated methionine in Drosophila
Stiebing et al[130]	2017	Spontaneous RS and SRS	d31-palmitic acid	C–D	Uptake and metabolic dynamics of d31-palmitic acid storage in human macrophages
Alfonso-García et al[131]	2016	SRS	D38-cholesterol	C–D	Imaging of intracellular cholesterol storage in mammalian cells
Villareal et al[132]	2016	SRS	Deuterated sterols	C–D	Imaging of intracellular localization of desmosterol
Li et al[133]	2014	SRS and Spontaneous RS	d7- glucose	C–D	Study of dynamic metabolism of glucose in single living cancer cells, visualizing

					de novo lipogenesis
Hu et al[134]	2014	SRS	d9- choline	C–D	Distribution of choline metabolites in cancer and non-cancer cells
Fu et al[135]	2014	Hypespectral SRS imaging	deuterated saturated and unsaturated fatty acid	C–D	Metabolic imaging of fatty acids in living C. elegans.
Yu et al[136]	2017	SRS with high throughput screening (HTS)	d34- oleic acid	C–D	Imaging of lipid synthesis in live C. elegans.
Zhang et al[137]	2011	Femtosecond excitation source with SRS imaging	d31- palmitic acid	C–D	3D sectioning of fat storage in live C. elegans
Wei et al[138]	2019	Clearing-enhanced DO-SRS microscopy	deuterium oxide	C–D	Volumetric (3D) chemical imaging of macromolecules in mouse tumors.
Chen et al[139]	2014	SRS and Spontaneous RS	^{12}C and ^{13}C isotopically edited alkynyl metabolic reporters	Alkyne	Multiplex chemical imaging of RNA, newly uptaken fatty-acids and DNA synthesis in live mammalian cells.
Hu et al[140]	2016	SRS	^{12}C and ^{13}C labeled EdU isotopologues	C–D, alkyne	Monitoring of DNA synthesis dynamics in mammalian hippocampal slice culture.
Shi et al[141]	2018	Spontaneous RS and SRS	deuterium oxide	C–D	In situ metabolic activities in animal tissue.
Zhang et al[142]	2019	Spontaneous RS and SRS	deuterated glucose	C–D	Imaging of glucose metabolism
Hong et al[143]	2018	SRS	deuterated glucose	C–D	Monitoring of glucose metabolism in a

					single living bacterium.
Yang et al[144]	2019	Spontaneous RS	Heavy water (D2O)	C–D	Antibiotic Susceptibility Testing of Pathogenic Bacteria
Zhang et al[145]	2017	SRS	d7- glucose, CD- amino acids, d31-palmitate acid, propargylcholine	C–D, alkyne	Cancer metabolism during the epithelial–mesenchymal transition of cancer cells
Shen et al[146]	2017	SRS	d-palmitate	C–D	Study of lipid metabolism in endoplasmic reticulum cell membrane.
Matthaeus et al[147]	2008	Spontaneous RS	deuterium-labelled liposomal drug carriers	C–D	Imaging of liposomal drug carrier systems inside cells.
Saar et al[148]	2010	SRS	deuterated ibuprofen	C–D	Ex vivo monitoring of drug delivery to mice ears.
Chen et al[149]	2015	Coherent Anti-Stokes Raman Scattering (CARS)	deuterated active molecules (glycerol, jasmonic acid and water)	C–D	Active molecule mapping inside artificial and human skin.
Saar et al[150]	2010	SRS	deuterated dimethyl sulfoxide (DMSO)	C–D	In vivo imaging of DMSO on a human forearm.

Table SII: Raman intensity versus EdU (RIE) and Raman shift of various moieties exhibiting a Raman signal in the cell- silent Raman region, as mentioned in studies of various Raman modalities.

**Compound**	**Molecular structure**	**Modality**	**Strength vs EdU**	**Raman Shift (cm^{-1})**	**Mean Raman shift (cm^{-1})**	**Ref**

Phenylalanine (Phe)	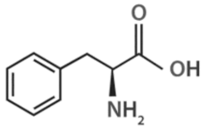	RS	0.2	1003	1003	30
Edu (Ethynyl-2'-deoxyuridine)	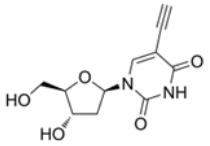	RS	1	2110	2110	30
Terminal Alkynes	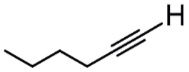	RS	0.485	2084-2113	2099	31
Silyl and Halo-alkynes	Ar—≡—X<br>R—≡—X	RS	1.25	2157-2195	2176	31
Internal alkynes	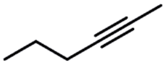	RS	3.17	2198-2248	2223	31
Diynes	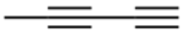	RS	3.4	2207-2249	2228	31
Bisaryl-substituted diynes (BADY)	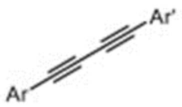	RS	25	2217-2221	2219	31
Nitrile	R−C≡N	RS	0.062	2242	2242	31
Azide	$N^{-}{=}N^{+}{=}N^{-}$	RS	0.022	2096	2096	31
MitoBADY	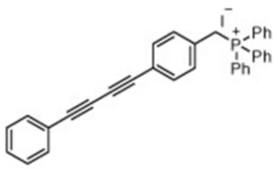	RS	27	2218	2218	32

Deuterated hexanoic acid with ^{11}CD	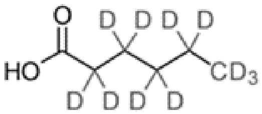	RS	0.062	2124	2124	31
Azobenzene (AERS)	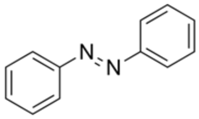	RS	104	2114-2222	2168	47
C–D	C–D	SRS	0.025	2100	2100	125
N-(3-azidopropyl)-4-(phenylbuta-1,3-diyn-1-yl)benzamide-phenyl ring	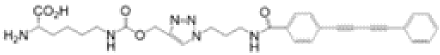	SRS	29	2225	2225	63
9CN-JCP	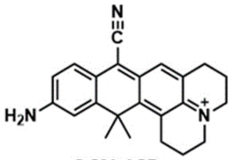 9CN-JCP	EPR - SRS	87	2220	2220	66
Poly(phenylene ethynylene)-PPE derivatives	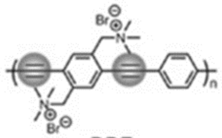 PPE	RS	1.8	2200	2220	116
PDDA	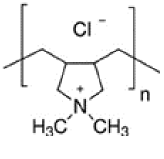	RS	200	2120	2120	118
Triple-bond-encoded polymer NPs	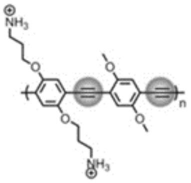	SRS microscopy	140	2223-2241	2232	117

